# The Self-Limiting Dynamics of TGF-β Signaling *In Silico* and *In Vitro*, with Negative Feedback through PPM1A Upregulation

**DOI:** 10.1371/journal.pcbi.1003573

**Published:** 2014-06-05

**Authors:** Junjie Wang, Lisa Tucker-Kellogg, Inn Chuan Ng, Ruirui Jia, P. S. Thiagarajan, Jacob K. White, Hanry Yu

**Affiliations:** 1 Computational and Systems Biology, Singapore-MIT Alliance, Singapore; 2 Mechanobiology Institute, Singapore; 3 Centre for Computational Biology, Duke-NUS Graduate Medical School, Singapore; 4 NUS Graduate School for Integrative Science and Engineering, National University of Singapore, Singapore; 5 Department of Physiology, National University of Singapore, Singapore; 6 School of Computing, National University of Singapore, Singapore; 7 Department of Electrical Engineering and Computer Science, Massachusetts Institute of Technology, Cambridge, Massachusetts, United States of America; 8 Department of Biological Engineering, Massachusetts Institute of Technology, Cambridge, Massachusetts, United States of America; The Centre for Research and Technology, Hellas, Greece

## Abstract

The TGF-β/Smad signaling system decreases its activity through strong negative regulation. Several molecular mechanisms of negative regulation have been published, but the relative impact of each mechanism on the overall system is unknown. In this work, we used computational and experimental methods to assess multiple negative regulatory effects on Smad signaling in HaCaT cells. Previously reported negative regulatory effects were classified by time-scale: degradation of phosphorylated R-Smad and I-Smad-induced receptor degradation were slow-mode effects, and dephosphorylation of R-Smad was a fast-mode effect. We modeled combinations of these effects, but found no combination capable of explaining the observed dynamics of TGF-β/Smad signaling. We then proposed a negative feedback loop with upregulation of the phosphatase PPM1A. The resulting model was able to explain the dynamics of Smad signaling, under both short and long exposures to TGF-β. Consistent with this model, immuno-blots showed PPM1A levels to be significantly increased within 30 min after TGF-β stimulation. Lastly, our model was able to resolve an apparent contradiction in the published literature, concerning the dynamics of phosphorylated R-Smad degradation. We conclude that the dynamics of Smad negative regulation cannot be explained by the negative regulatory effects that had previously been modeled, and we provide evidence for a new negative feedback loop through PPM1A upregulation. This work shows that tight coupling of computational and experiments approaches can yield improved understanding of complex pathways.

## Author Summary

TGF-β signaling pathway regulates a variety of cellular responses, such as differentiation, migration and apoptosis. Phosphorylated R-Smad, the central signaling protein in this pathway, exhibits self-limiting behaviors: it not only decreases quickly after TGF-β is removed, but it also decreases slowly when TGF-β remains abundant. These two self-limiting behaviors are important to understand clearly because diseases such as cancer and fibrosis might benefit from treatments to decrease Smad signaling. Several negative regulatory effects have been reported previously, and we studied the dynamics of these effects with computational modeling. Analyzing the timing of negative regulation revealed that the three most widely accepted effects were not sufficient to explain the observed declines. After considering and excluding several alternative models, we arrived at a model in which TGF-β upregulated the phosphatase PPM1A. We tested for PPM1A upregulation in cell culture experiments. In addition, our model was able to explain why different durations of TGF-β exposure could cause seemingly opposite results about the importance of Smad degradation.

## Introduction

Transforming Growth Factor-β (TGF-β), a regulator of cell migration and cell fate, is a pharmaceutical target for the treatment of metastatic cancer and fibrotic diseases [1]. Signal transduction from extracellular TGF-β to the cell nucleus through the Smad pathway is well documented [2]–[7]. The TGF-β ligand binds sequentially to the type II TGF-β receptor, a constitutively active kinase, and then to the type I receptor, to form a ligand-receptor complex (LRC). The type I receptor is activated by the type II receptor and then phosphorylates the R-Smads (Smad2 and Smad3) at two C-terminal serine residues. Upon phosphorylation, R-Smads form a homomeric complex or a heteromeric complex with Co-Smad (Smad4). The key outcome of the Smad cascade is the accumulation of phosphorylated R-Smad (phospho-R-Smad) in the nucleus, affecting the transcriptional regulation of many genes[7], [8].

Smad signaling is known to decrease quickly after TGF-β stimulation, causing rapid decline of phospho-R-Smad after its initial peak. The HaCaT cell line has been adopted as an experimental model system for quantifying the detailed signaling of the TGF-β/Smad system. In HaCaT cells, there is a rapid decline of phospho-R-Smad after short exposure to TGF-β stimulation (30–45 min) [9], [10], and a gradual decline after long exposure to TGF-β (6–24 hr) [9], [11]. The duration of phospho-R-Smad activation could be crucial for regulation of different genes [12]. The self-limiting behavior of Smad signalling (i.e., negative regulation) may be caused by ligand-induced receptor inhibition [13]–[19], phospho-R-Smad dephosphorylation [9], phospho-R-Smad degradation [11], [20]–[23], or other effects. Extensive experimental evidence has documented multiple modes of negative regulation, but the relative roles and combined effects are not well understood.

Previous computational models of TGF-β/Smad signaling have contributed important biological insights, but they have only simulated some selected negative regulatory effects. Vilar *et al*. built a model of TGF-β receptor trafficking dynamics, including ligand-induced receptor degradation, which was able to simulate some key dynamic observations such as the peak and decline of phospho-R-Smad levels [24]. Models by Klipp and co-workers extended the work of Vilar *et al*. to include Smad phosphorylation and nuclear translocation [25], and to include transient versus sustained Smad signaling [12]. These models used simple representations for negative regulation, and gave a strong role to receptor degradation. The model by Schmierer *et al*. provided important insights into the Smad nucleo-cytoplasmic shuttling [10], but the only negative regulatory effect in this model was dephosphorylation. Other modeling studies have focused on robustness and *in silico* perturbation analysis [26], [27]. Mathematical models have yielded important insights, but they have not represented TGF-β/Smad negative regulation with enough detail for analyzing the contributions of different negative regulatory effects, nor for evaluating alternative hypotheses.

In this work, we developed a series of computational models, representing individual and combination effects of R-Smad negative regulation. Comparisons between models and observations revealed negative regulation to occur at more than one time-scale. We classified negative regulatory effects into fast-mode (5–240 min) and slow-mode (1–24 hr), depending on how quickly they act (and how quickly they equilibrate to steady state). Models then showed that at least one fast-mode and one slow-mode effect would be required for a model to fit the phospho-R-Smad dynamics in both short-exposure and long-exposure experiments. R-Smad Dephosphorylation was a fast-mode effect and it was strong enough to explain the fast-mode observations. Receptor Degradation and P-Smad Degradation were slow-mode effects, but they were too weak to explain the observed slow-mode decline. With a shortfall in explaining why R-Smad continues to decline hours after TGF-β stimulation, we sought a novel slow-mode effect. A second key finding of this work is a novel negative feedback effect, confirmed experimentally, in which the phosphatase PPM1A is upregulated after TGF-β stimulation. A final model hypothesizes how PPM1A might be upregulated with delayed activity, based on previously published molecular mechanisms for regulating PPM1A degradation [28], [29]. Another final contribution we provide is an explanation for a previous controversy about proteasomal degradation of phospho-R-Smad [9], [11], [20], [21]. Previous experiments inhibiting proteasomal degradation showed either strong effects [11], [20], [21] or no effects [9] on phospho-R-Smad levels. These seemingly contradictory trends were both mathematically consistent with our model, and the disparity could be explained by different durations of TGF-β exposure.

## Results

**Figure 1:**
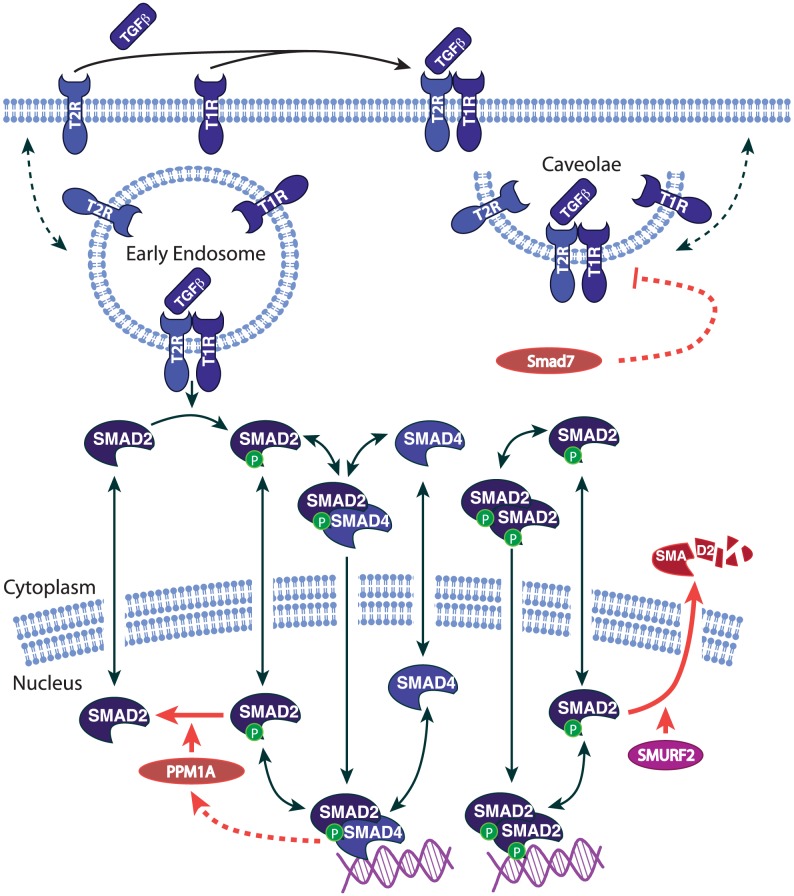
The pathway diagram of Smad signaling (using symbols from BioCarta). The dashed arrows indicate those reactions which are modeled in black box. The red arrows indicate the negative regulatory effects: (1) PPM1A dephosphorylating phospho-R-Smad; (2) Smurf2 induced proteasome degradation of phospho-R-Smad; (3) I-Smad induced receptor inhibition; (4) PPM1A upregulation by Smad signaling.

A series of computational models were constructed (Table 1) examining negative regulatory effects in TGF-β/Smad signaling. All models share a common skeleton of Smad signaling (Figure 1), with TGF-β receptor internalization and trafficking, and Smad nucleo-cytoplasmic shuttling, based on previous models [10], [24], [25]. Molecular interactions were modeled using ordinary differential equations (ODEs) for mass action kinetics, and systems of ODEs were simulated using KroneckerBio [30] in Matlab (Mathworks, Natick, MA). The goodness-of-fit of parameter estimation was evaluated by the sum of squared errors (Text S7). The HaCaT cell line was used in our biological assays. Western blots and ELISA were used for time-series measurements of protein levels. Complete model specifications, parameter estimation, as well as biological experiments are specified in Materials and Methods and Supporting Information. In computational models and biological experiments, we used phosphorylated Smad2 (P-Smad2) to represent all phospho-R-Smad species, because P-Smad2 and P-Smad3 have highly similar dynamics in HaCaT cells [9].

**Table 1:** A summary of the 8 models, showing the models with negative regulatory effects.

Negative Regulatory Effect	Model Number							
	1	2	3	4	5	6	7	8
R-Smad Dephosphorylation	+	+	-	-	+	+	+	+
Receptor Degradation	-	+	+	-	+	-	-	-
P-Smad Degradation	-	-	-	+	+	+	+	+
Endogenous Synthesis and Degradation of R-Smad	-	-	-	-	-	-	+	+
PPM1A Stabilization	-	-	-	-	-	-	-	+
Which dataset caused rejection of the model:	pS	T1R	pS	pS	tS	tS	pS	None

The + sign indicates that a negative regulatory effect is included in a model, and - indicates a negative regulatory effect is NOT included.

pS: phospho-R-Smad time series.

T1R: type I receptor time series.

tS: total R-Smad time series.

### Modeling the Negative Regulatory Effects

Smad signaling is enormously complex, as proven by a vast literature of previous work. We first considered negative regulatory effects from the published literature, for the purpose of selecting a set of effects relevant to our studies.

#### (1) Receptor Degradation

Smad7 (Inhibitor Smad or I-Smad) can target the ligand-receptor complex for degradation by recruiting E3-ligases [17]–[19]. In many cell types, Smad complexes can also induce the production of Smad7 (I-Smad), as a form of negative feedback [13], [14]. However, in HaCaT cells, Smad7 levels are high and have minimal change after TGF-β stimulation [31]. Thus, we modeled Receptor Degradation as a first-order degradation reaction induced by a high constant level of Smad7. For comparison, we did try simulating a non-HaCaT model with Smad7 feedback, but the results were very similar to our model with a high constant level of Smad7 (data not shown). In addition to receptor degradation, there are two additional ways that I-Smad can antagonize the ligand-receptor complex: (a) It can block the activation site of the receptor kinase; and (b) It can recruit PP1c to dephosphorylate the type I receptor kinase [32]. We simulated these variants of the effect in Supporting Information (Text S2). In summary, we model Receptor Degradation as an intrinsically active process, but its target substrate is an activated species (the ligand-receptor complex), meaning that the reaction proceeds only when the target species is available due to TGF-β stimulation.

#### (2) P-Smad Degradation

R-Smad can be phosphorylated at its tail region or its linker region. We use the term “phospho-R-Smad” to refer to tail-region phosphorylation, which is more important for gene regulatory function [21] than the linker-region phosphorylation. Phosphorylation of nuclear R-Smad at its linker region causes Smurf2 to target R-Smad for proteasome-dependent degradation [11], [21]–[23]. Assuming Smurf2 concentration to be constant, and assuming linker-region phosphorylation to be proportional to tail-region phosphorylation [21], we set the rate of R-Smad degradation to be proportional to the concentration of nuclear R-Smad. Again, this is an intrinsically active process, but its target is an activated (phosphorylated) species, so the process only occurs when the target species has been activated by TGF-β stimulation.

#### (3) R-Smad Dephosphorylation

Phospho-R-Smad in the nucleus is dephosphorylated at the tail region specifically by PPM1A, a member of the PP2c family [9]. The rate of dephosphorylation is modeled to be proportional to the concentration of phospho-R-Smad in the nucleus. As previously, this is an intrinsically active process that only targets the activated species.

#### (4) Steady-state effects

Many factors have been found to regulate the gene expression, localization, degradation, or post-translational modification of Smad system proteins. A feedback loop is formed whenever these upstream factors are regulated by TGF-β. In contrast, if the upstream factors are independent of TGF-β (meaning constant during the course of a TGF-β-stimulation experiment), then we call them steady-state effects. Steady-state effects can alter the resting levels (the “initial concentrations”) of the Smad system, and they can alter the absolute magnitude of phospho-R-Smad activation, but they do not explain the declining slope of phospho-R-Smad after TGF-β activation, because steady-state effects do not change during TGF-β stimulation. Because we study the decline rather than the absolute activation of phospho-R-Smad, we will not consider steady-state effects unless otherwise specified.

Any negative regulatory effect that was identified without TGF-β stimulation, we assumed to be a TGF-β-independent, steady-state effect. This assumption is not necessarily correct because biological networks often show greater interconnectivity than anticipated. Regulatory influences that we categorized as “steady-state effects” included degradation of Smad4 [33], [34]; and sequestration of Smads [35]–[39].

#### (5) PPMIA stabilization

The PPM1A phosphatase can be protected from proteasomal degradation by binding PTEN [28], meaning that PTEN can serve as a negative regulator of Smad signaling. However, this effect had never been found to occur in response to TGF-β stimulation. In fact, the contrary was found. In the fibroblast cells where PTEN-mediated stabilization was observed, the stabilization was actually shut off by TGF-β stimulation [28], meaning that alteration of PPM1A stability contributed to self-perpetuation of TGF-β/Smad signaling, rather than providing a self-limiting mechanism. Although PTEN is a negative regulator of Smad signaling, it was found participating in a positive (double negative) feedback loop. Therefore, we assumed initially that altered stability of PPM1A was not contributing to the decline of Smad phosphorylation after TGF-β treatment.

#### (6) Receptor Internalization

Receptor Internalization was also included in all models, but we did not consider receptor internalization to be a negative effect, for saturating doses of TGF-β and for the 0.5–24 hr timescale. For additional explanation, see Text S1.

#### (7) Gene regulation and downstream effects

The gene regulatory functions of phospho-R-Smad are the result of complex interactions with many factors. Some proteins interfere with the interaction between the Smad complex and transcription factors, co-activators, or with the recruitment of HDAC [40], [41]. These effects, although crucial for the gene regulatory functions of Smad signaling, are downstream of phospho-R-Smad. Our datasets measure phospho-R-Smad and our models study upstream events that regulate phospho-R-Smad levels. Thus, gene regulation and downstream effects are beyond the scope of our models.

#### (8) Sequestration of Smad by SnoN

R-Smad can be sequestered by cytoplasmic SnoN [42]. Although SnoN effects may be significant in many cell types, cytoplasmic SnoN is not detectable in HaCaT [43]. Therefore, we did not consider sequestration of Smad by SnoN in our modeling.

In summary, the most compelling negative regulatory effects in HaCaT appeared to be (1), (2), and (3) above, illustrated in Figure 1. An additional self-limiting effect shown in Figure 1 is a negative feedback loop, described later, involving PPM1A upregulation.

### Negative Regulation Occurs at Multiple Time Scales

R-Smad dynamics depend on the duration of TGF-β stimulation. When TGF-β is administered in excess (2 ng/ml) [44], [45] for 24 hrs, phospho-R-Smad peaks at about 1 hr and then decays for 24 hrs [9]. When TGF-β is administered for 30 min and then removed (by washing following by receptor inhibition with the compound SB-431542), phospho-R-Smad is eliminated within 4 hrs [9], [10].

**Figure 2:**
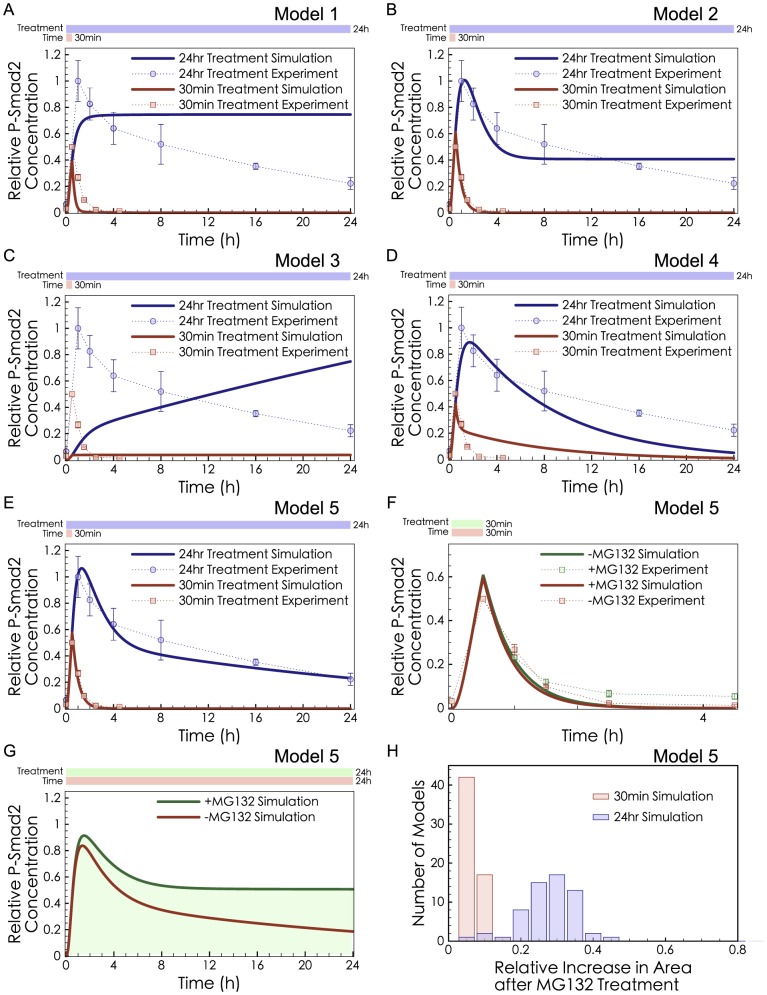
Model fitting results with different combinations of known negative regulatory effects. (**A–E**) Dots: experimental data from Lin *et al*. [9]. All P-Smad2 measurements used total cell lysate. Curves: the model simulations were fitted to the two sets of data simultaneously. (**A**) Model 1: R-Smad Dephosphorylation; (**B**) Model 2: R-Smad Dephosphorylation and Receptor Degradation; (**C**) Model 3: Receptor Degradation; (**D**) Model 4: P-Smad Degradation; (**E**) Model 5: R-Smad Dephosphorylation, Receptor Degradation and P-Smad Degradation. The reactions of each model are listed in the Supporting Information. (**F–H**) Predictions of the best-fit model (Model 5) in MG132 pre-treated cells. Simulation of MG132 treatment was performed by turning off the Smurf2-induced P-Smad Degradation (setting *kdeg_pSmad2_* = 0) in Model 5. (**F**) Comparison of the model prediction and experimental data from Lin *et al*. [9] in the short-exposure experiment. (**G**) Model prediction in the long-exposure experiment. The green shaded area shows the cumulative difference between +MG132 and -MG132. (**H**) A histogram plots the cumulative differences seen in the short-exposure experiment (red) and the long-exposure experiment (blue).

Our first modeling studied the kinetics of the three negative regulatory effects selected from the literature review. We were curious whether they would have different kinetic implications for the system. Both short-exposure and long-exposure TGF-β treatment datasets (Figure 2) were utilized when building the models of negative regulation (Table 1, Table S2). The models were simulated to obtain the dynamics of their effects and to estimate their potential contributions to the down-regulation of phospho-R-Smad (0.5-24 hr).

Model 1, with R-Smad Dephosphorylation, was able to recapitulate the short-exposure TGF-β treatment experiment, as dephosphorylation is a fast process. This dephosphorylation model could turn off the signal once the stimulus was cut off (Figure 2A red curve), but it reached a steady state at about 1 hr and was not able to recapitulate the extended 24 hr decline of phospho-R-Smad in long-exposure TGF-β treatment (Figure 2A blue curve). Thus we describe R-Smad Dephosphorylation as a “fast-mode” effect. To explain the prolonged decline during long-exposure experiments, a complementary “slow-mode” might be provided by cumulative processes such as degradation. Model 2 combines Receptor Degradation and R-Smad Dephosphorylation. It succeeded in recapitulating the short-exposure TGF-β treatment very well, and it had moderately good agreement with the long-exposure dataset (Figure 2B). As a control, we modeled Receptor Degradation alone (Model 3), but it could not provide an early decline in the short-exposure experiment (Figure 2C). Thus, Receptor Degradation serves as a slow-mode effect as it was able to explain the gradual and protracted decline of phospho-R-Smad in the long-exposure experiment but not the steep decline of phospho-R-Smad in the short-exposure experiment. Another cumulative process of decline is P-Smad Degradation. A model with P-Smad Degradation alone (Model 4) achieved significant negative regulation for the long-exposure case (Figure 2D), because P-Smad Degradation would persist for many hours. However, Model 4 had difficulty explaining both the short-exposure and long-exposure datasets simultaneously. If P-Smad Degradation is strong, it could recapitulate the steep decline after short-exposures, and if it is weak effect, it could recapitulate the gradual decline after long-exposures. Since it cannot be both strong and weak, it cannot explain both behaviors. Note that previous experimental evidence showed that P-Smad Degradation is not responsible for fast-mode effects in short-exposure conditions [9]. Having simulated each of the three negative regulatory effects in isolation, we could conclude that no single negative regulatory effect was able to explain phospho-R-Smad dynamics. We infer that the experimentally observed levels of phospho-R-Smad arise from a combination of fast-mode and slow-mode effects (or from higher-order combinations of effects).

Many models have omitted P-Smad Degradation from simulations [10], [12], [24], [25], perhaps because this effect was found to be insignificant in the experiments of Lin *et al*. [9]. Noting that the Lin experiments used short-exposure conditions, we asked whether P-Smad Degradation, a slow-mode effect, might have greater significance during the negative regulation induced by long-exposure treatments. Model 5 incorporated R-Smad Dephosphorylation, Receptor Degradation, and P-Smad Degradation (Figure 2E). P-Smad Degradation was significant in this model (Figure 2F-G) when its impact was measured after more than 1 hr of TGF-β treatment. We also fitted a variety of models to the short-exposure and long-exposure experiments. The cumulative difference in phospho-R-Smad between +MG132 and -MG132 was minor in the short-exposure experiment and significant in the long-exposure experiment (Figure 2H). As yet, we have no basis for knowing which type of slow-mode degradation would be most important in R-Smad signaling.

### Receptor Degradation Is Not Supported by Experiments

**Figure 3:**
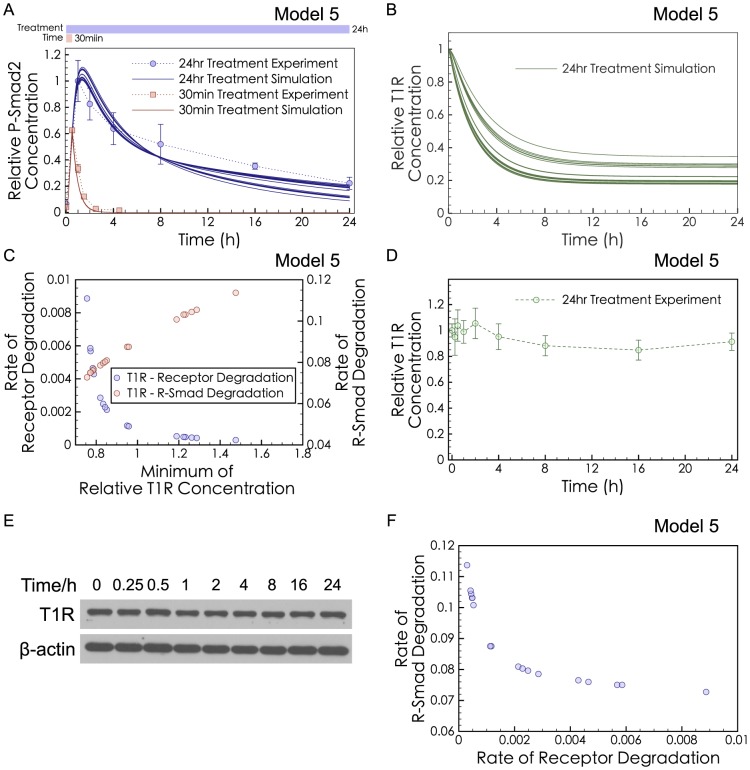
Predictions and validations of Receptor Degradation. (**A**) Different rates of I-Smad-induced Receptor Degradation (*klid* = 10^−6^∼10^−2^) were applied to Model 5, and the rate of Smurf-induced P-Smad Degradation (*kdeg_pSmad2_*) was fitted to the short-exposure experimental data (red dots) and the long-exposure experimental data (blue dots). All the other parameters were kept the same as those in Model 5 (**B**) Different Receptor Degradation rates led to different levels of the type I receptor (T1R). Green curves were generated from all models in panel (A) with *klid* = 10^−6^∼10^−2^ and *kdeg_pSmad2_* estimated. (**C**) In the fitted models in panel (A), the T1R level has negative correlation with the Receptor Degradation rate (*klid*) but positive correlation with the P-Smad Degradation rate (*kdeg_pSmad2_*). (**D**) Quantified data from 3 replicates of the western blot in (E). There is no significant loss of the T1R comparing the first and last data points (P>0.05). (**E**) Western blot of the T1R from whole cell lysates of HaCaT cells treated with TGF-β for 24 hrs (representative of 3 replicates). (**F**) In the fitted models in panel (A), the rates of Receptor Degradation (*klid*) and P-Smad Degradation (*kdeg_pSmad2_*) have negative correlation.

**Figure 4:**
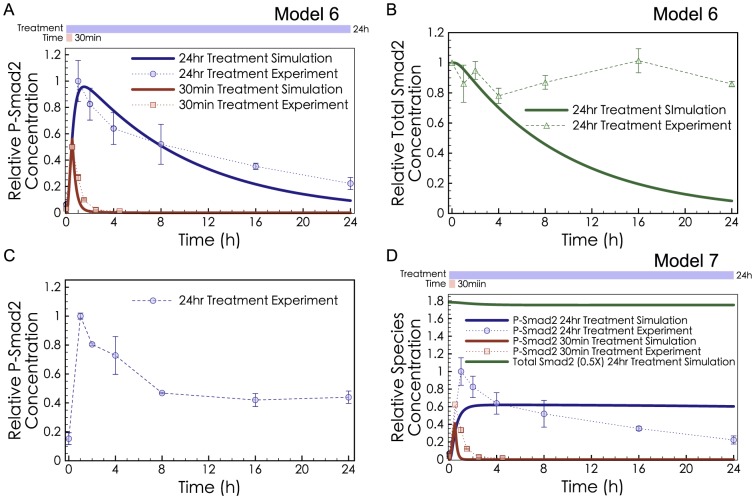
Simulations and experiments for P-Smad Degradation. (**A**) Model 6 with P-Smad Degradation and R-Smad Dephosphorylation (but no Receptor Degradation) was fitted to both the short-exposure (red) and long-exposure (blue) experimental data. (**B**) Model 6 predicted significant loss of total R-Smad (green curve), while ELISA measurements showed insignificant change (P>0.05, comparing the first and last data points) in total R-Smad concentration (green dots). (**C**) ELISA measurements of phospho-R-Smad are consistent with previous measurements performed by Western blot [9]. Cell lysates were from the same samples as panel B. (**D**) Model 7 was fitted to the phospho-R-Smad data while constraining the total R-Smad level to be constant.

We next tried to assess the relative impact of two slow-mode effects, Receptor Degradation and P-Smad Degradation, on the dynamics of phospho-R-Smad in long-exposure TGF-β treatment. The rate constant for Receptor Degradation and the rate constant for P-Smad Degradation were varied *in silico* (Figure 3A), showing that many ratios were equally good at fitting the observed dynamics. Several of the successful models exhibited a strong decline in T1R, the type I receptor (Figure 3B). Moreover, the degree of T1R decline was correlated with the rate of Receptor Degradation and the rate of P-Smad Degradation (Figure 3C). Thus, to quantify the relative contribution of Receptor Degradation and P-Smad Degradation in HaCaT cells, we measured T1R experimentally at 9 time points (from 15 min to 24 hr) after TGF-β stimulation (with n = 3 replications and significance determined by Student's t-test). Surprisingly, there was no significant loss of T1R (type-I receptor) observed in experiments (Figure 3D–E), even at late time points. (As positive control, phospho-R-Smad time series concentrations were measured in Figure 4C). Previous work has already shown that T2R (type II receptor) shows no decrease after 2 ng/ml of TGF-β treatment in HaCaT cells [46]. Unchanged receptor levels indicate that Receptor Degradation is very weak in HaCaT cells. A weak role for Receptor Degradation has also been suggested by the experimental work of Clarke *et al*. [47]. Other forms of receptor inactivation or sequestration may occur without changing the total T1R concentration, but there is less published evidence for these possibilities (modeling analysis rejected these possibilities as well, in Text S2). Note that the set of models (Figure 3A) capable of explaining the dynamics of phospho-R-Smad decline all exhibited a negative correlation between the degree of Receptor Degradation and the degree of P-Smad Degradation (Figure 3F), suggesting that these two effects would be balanced alternatives. In light of our experimental finding that Receptor Degradation is a very weak effect, we next turned to P-Smad Degradation as the alternative slow-mode effect to explain the long-term decline of phospho-R-Smad.

### P-Smad Degradation Is Not Sufficient to Explain the Peak and Decline of Phosphorylated R-Smad

A model with R-Smad Dephosphorylation and P-Smad Degradation (Model 6, without Receptor Degradation) provided an excellent fit to both the long-exposure and short-exposure treatment data (Figure 4A). However, an unavoidable consequence of this model was dramatic decline of total R-Smad (Figure 4B). Previous experiments in HaCaT cells failed to observe a large fold-change of total R-Smad [9] but the amount of decline was not quantified. To clarify this potential conflict, we repeated the experimental measurement of total R-Smad levels after TGF-β treatment, using ELISA assays, a more quantitative method. Measurements of total R-Smad at 7 time points during 24 hrs of TGF-β treatment showed no significant decrease of total R-Smad (Figure 4B–C). There is an apparent conflict between the constant level of total R-Smad (observed experimentally) and the significant degradation of R-Smad induced by TGF-β (according to Model 6).

Degradation might be more difficult to rule out if we consider TGF-β-stimulated degradation in combination with **Endogenous Synthesisand Degradationof R-Smad**. If endogenous R-Smad is synthesized in an unphosphorylated form, and targeted by Smurf2 for degradation only in its phosphorylated form, then can P-Smad Degradation explain the decline of phospho-R-Smad despite the constant levels of total R-Smad? We therefore expanded the model to include Endogenous Synthesis and Degradation of R-Smad (Model 7). However, Model 7 diverged strongly from the observed dynamics of phospho-R-Smad, when constrained to maintain a constant level of total R-Smad.

To summarize these results, P-Smad Degradation can only affect the shape of the phospho-R-Smad curve if it is not balanced by synthesis, in which case it would cause an unrealistic decline in the total Smad levels. If P-Smad Degradation is balanced by Smad synthesis, then it can only affect the height but not the shape of the phospho-R-Smad curve. Therefore we can rule out strong P-Smad Degradation (not balanced by synthesis) as an explanation for the later decline in the phospho-R-Smad curve shape. We cannot rule out the presence of significant P-Smad Degradation accompanied by Smad synthesis.

Hence, our model-driven experimental tests, sensitivity analysis (Text S3), and modeling analysis showed that P-Smad Degradation and Receptor Degradation were not sufficient to explain the 1–24 hr decline in phospho-R-Smad dynamics. We next sought some other negative regulatory effect that could help explain the peak and decline of phospho-R-Smad after a long exposure to TGF-β.

### PPM1A Is Upregulated after Treatment with TGF-β

**Figure 5:**
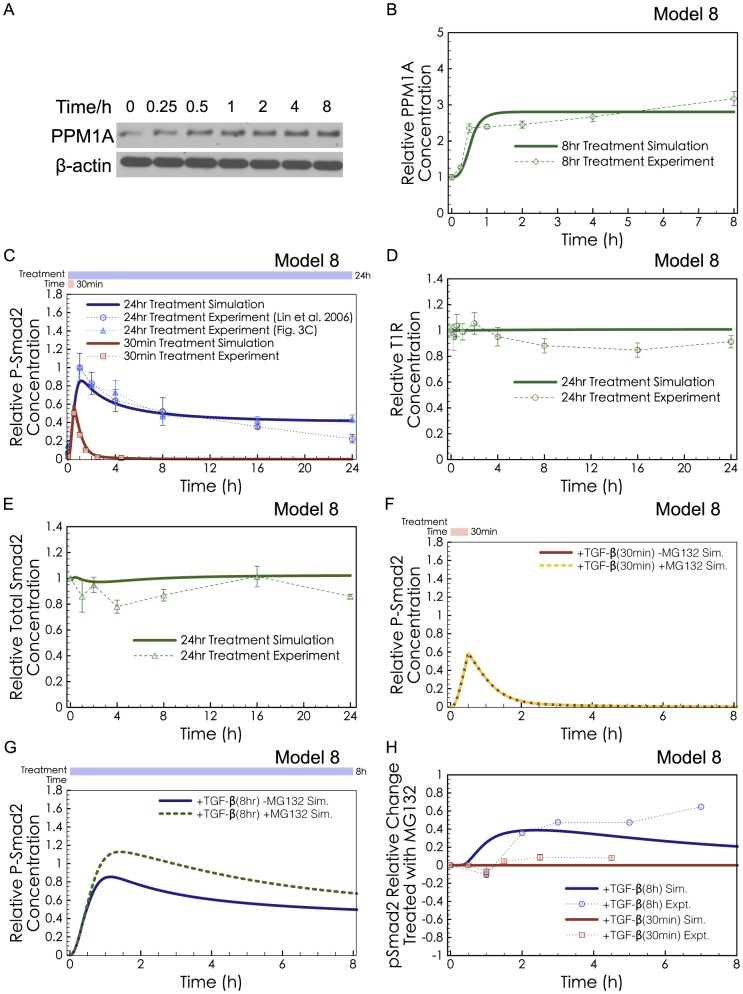
Predictions and validation of PPM1A Upregulation. (**A**) Western blot of PPM1A in HaCaT cells with 2 ng/ml TGF-β treatment, representative of 3 replicates. (**B**) Model 8 predicted PPM1A upregulation under long-exposure of TGF-β (green curve). Our experimental validation showed significant upregulation of PPM1A (green dots, quantification from 3 Western blots, P<0.05 comparing the untreated data point and the 1 hr data point). (**C**) Model 8 was fitted to the long-exposure and the short-exposure phospho-R-Smad experimental data. (**D**) Model 8 predicted unchanged T1R levels (green curve), in agreement with our experimental results (green dots). (**E**) Model 8 predicted unchanged total R-Smad levels (green curve), in agreement with our experimental results (green dots). (**F**) Red solid curve shows simulation of Model 8 with short-exposure (30 min) of TGF-β, while the yellow dotted curve shows the same simulation except with MG132 pre-treatment. MG132 was simulated as turning off Smurf2-induced P-Smad Degradation (*kdeg_pSmad2_* = 0), but having no impact on basal degradation of Receptors, unphosphorylated R-SMAD, or PPM1A. (**G**) The blue solid curve shows simulation of Model 8 with long-exposure (8 hr) of TGF-β, and the green dotted curve shows the same simulated except with MG132 pre-treatment. (**H**) The relative change in P-Smad2 levels after MG132 treatment, calculated from Eq. 1 and simulations of Model 8. The P-Smad2 change simulated using Model 8 in both short-exposure (30 min, red curve) and long-exposure (8 hr, blue curve) simulations was compared with the P-Smad2 changes in the experimental results of Lin *et al*. [9] (30 min-exposure, red dots) and Alarcon *et al*. [21] (6 hr-exposure, blue dots). Data points from Alarcon *et al*. [21] were quantified from one published image. The discrepancy between our simulations and Alarcon *et al*. for the 7 hr measurement may be partially explained by MG132-independent differences. Their -MG132 control decreases much faster than that from Lin *et al*. [9] and from our experiments.

After excluding the three well-accepted effects of Smad negative regulation, we then examined possible alternative influences at different steps along the Smad pathway, seeking quantitative consistency with the observed peak and decline of phospho-R-Smad. One scenario that could not be rejected on kinetic grounds was upregulation of PPM1A, the phosphatase targeting phospho-R-Smad. If PPM1A were to be upregulated by TGF-β signaling, this could help explain the decline of phospho-R-Smad after long exposure to TGF-β (Text S4). To test this possibility, we performed Western blots of the PPM1A protein after TGF-β treatment. HaCaT cells were treated with 2 ng/ml of TGF-β and measured after 0.25, 0.5, 1, 2, 4, or 8 hr. We found that the intensity of the PPM1A western blot band increased 2.4-fold after 1 hour of TGF-β treatment (p<0.05, Figure 5A-B). To the best of our knowledge, this is the first study to report that TGF-β causes upregulation of the PPM1A phosphatase.

The increased abundance of PPM1A after TGF-β stimulation could be due to some type of decreased degradation and/or increased production. To aid future studies in investigating how the upregulation occurs, we have constructed a hypothetical mechanism, PPM1A Stabilization, in which we speculate that PTEN may be involved. Model 8 includes PPM1A Stabilization plus all the mechanisms of Model 7 (R-Smad Dephosphorylation, P-Smad Degradation, and Endogenous Synthesis and Degradation of R-Smad). Text S5 provides a full specification of Model 8.

In previous studies, Bu et al. found that PTEN can bind to PPM1A and protect it from degradation [28]. These studies of PPM1A stability occurred in fibroblasts, where TGF-β caused dissociation of PTEN and PPM1A, leading to downregulation, not upregulation of PPM1A. In other words, they found PTEN to be a negative regulator of Smad signaling, but in their fibroblasts, TGF-β decreased this negative effect causing self-perpetuation (positive feedback) rather than self-limitation (negative feedback) of the Smad signal. The binding of PTEN in response to TGF-β is known to differ between fibroblasts and HaCaT keratinocytes. Hjelmeland *et al*. found that in HaCaT cells, TGF-β stimulation caused formation of a PTEN-Smad complex [29], not dissociation of the PTEN complex [28]. They did not measure participation of PPM1A in that complex, but based on our analysis of the trends from [28] and [29], we propose a scaffolding role for phospho-R-Smad to promote association between PTEN and PPM1A in HaCaT cells. In other words, Model 8 speculates that TGF-β stimulation would induce PTEN association to stabilize PPM1A. This implies that there is some new or unknown mechanism upstream of PTEN, to explain why TGF-β signaling would promote PTEN-PPM1A association in one cell type and dissociation in another cell type. Model 8 assumes that PTEN and PPM1A would have a low on-rate for binding each other in HaCaT cells without phospho-R-Smad, but they would readily form a ternary complex in the presence of phospho-R-Smad. Thus, PPM1A would not be strongly stabilized in unstimulated HaCaT cells. After TGF-β stimulation, the phospho-R-Smad mediated association between PTEN and PPM1A would protect PPM1A from degradation and create negative feedback in the system. Note that Model 8 does not imply any alteration of total PTEN protein levels, merely the recruitment of PTEN by phospho-R-Smad into complexes with PPM1A. Simulations of Model 8 in Supplementary Figure S9 confirm that total PTEN levels could in theory remain constant (as observed in [29]) while levels of the PTEN-PPM1A complex could change over time.

Simulations of Model 8 were consistent with all the observed dynamics for the impact of TGF-β on HaCaT cells. This model was sufficient to explain the complete dynamics of phospho-R-Smad after short or long exposures to TGF-β (Figure 5C), the dynamics of PPM1A (Figure 5B), and the unchanged levels of T1R and total R-Smad (Figure 5D-E). With the key experimental trends satisfied, we next tested Model 8 against another dataset, obtained from combination treatment with TGF-β and a chemical inhibitor MG132.

Previous studies assessed P-Smad Degradation using MG132 to inhibit proteasomal degradation, but with conflicting conclusions: Massague *et al*. saw a strong impact, implying an important role for degradation [11], [21], while Lin *et al*. found negligible impact from MG132 [9]. Both protocols measured the long-term dynamics of phosphorylated Smad2, but the Lin protocol triggered phosphorylated Smad2 using a 30 min exposure to TGF-β, while the Massague protocol used a 6 h exposure. Simulations of Model 8 with MG132 inhibition of proteasomal degradation show that MG132 would have minimal impact on Smad signaling, when triggered by short exposure to TGF-β (Figure 5F). In surprising contrast, MG132 would have a strong impact on Smad signaling, when phospho-R-Smad is triggered by longer exposures to TGF-β (Figure 5G). Figure 5H compares the P-Smad2 Change calculated from Figure 5F (red curve) and Figure 5G (blue curve) with experimental data from Lin *et al*. (Figure 1C in [9]) (red dots) and Alarcon *et al*. (Figure 2G in [21]) (blue dots). The P-Smad2 Change was calculated as Eq. 1.
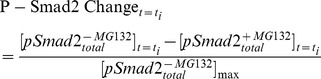
(Eq.1)


Model 8 shows, mathematically, that the Lin observations and the Massague observations can be generated from the same system. Model 8 contains hypothetical mechanisms (e.g., PPM1A Stabilization) and imperfect parameters (e.g., reaction rate constants), but it suffices to prove that the seeming conflict between Lin et al. and Massague et al. is not necessarily a contradiction. In summary, the combination of several negative regulatory effects was consistent with, and sufficient to explain, the observed nuances of negative regulation and degradation in the Smad signaling system.

## Discussion

Several negative regulatory effects in the Smad signaling pathway have been identified and individually studied [9], [11], [13], [14], [17], [21], [32], [48]. We focused our modeling and experiments on these specific effects with published evidence. R-Smad Dephosphorylation by PPM1A is widely recognized to be a strong form of negative regulation, having significant fast-mode impact. However, the known slow-mode effects could only recapitulate phospho-R-Smad dynamics at the expense of very strong, cumulative degradation; as much as 90% decrease of T1R at 24 hr (Figure 3B), or 90% decrease in total R-Smad at 24 hr (Figure 4B). Our experimental measurements in HaCaT found that total T1R protein levels did not decline significantly (Figure 3B, 3D), nor did total R-Smad (Figure 4B). This contrasts with previous work in 293T and COS-1 cells [17], [19]. In [17], 293T cells were transfected with I-Smad which was able to induce significant receptor degradation. The significant degradation seen in [17] may be due to transfection [47] or may be due to cell line differences. Although most dynamic models of signal transduction represent an amalgam of findings from multiple cell lines, our model (and the previous models we rely on) are specific to the HaCaT cell line. Thus a discrepancy with [17] is not necessarily a flaw of our model.

**Figure 6:**
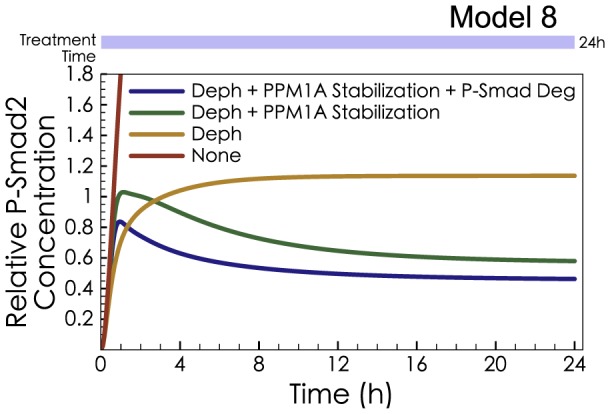
Relative contributions of different negative regulatory effects in our final model (Model 8). The effects of R-Smad Dephosphorylation, PPM1A Stabilization and P-Smad Degradation were removed one after another from Model 8, and the dynamics of P-Smad2 were simulated after 24 hr TGF-β treatment. In the absence of any negative regulatory effect (red curve), P-Smad2 levels climbed rapidly beyond the scale of the plot; and are not shown for time points after 1 hr.

In light of our experimental measurement that TGF-β treatment does not cause any significant drop in total R-Smad levels, and the evidence showing no significant decline in type I or type II receptor levels, we conclude that degradation effects, if they occur in HaCaT, must be counterbalanced by endogenous synthesis. Model 7 simulated a balance of synthesis and degradation (Endogenous Synthesis and Degradation of R-Smad) such that phospho-R-Smad was degraded while unphosphorylated Smad was synthesized; this model was not able to induce the observed decline of phospho-R-Smad in long-exposure experiments. The first key contribution of our work was to conclude that degradation of R-Smad or T1R, with or without endogenous synthesis, is not sufficient to explain the slow-mode of Smad negative regulation in HaCaT cells. Degradation with synthesis remains a plausible effect of negative regulation, but it must occur alongside other effects. Figure 6 shows the relative contributions of different negative regulatory effects in our final model (Model 8): R-Smad Dephosphorylation was crucial for maintaining a limited level of phospho-R-Smad (compare red versus yellow curves); PPM1A Stabilization was capable of explaining the decline after the peak of phospho-R-Smad (compare yellow versus green curves); and P-Smad Degradation could further adjust the absolute level of phospho-R-Smad (compare green versus blue curves).

The second key contribution of our work was the discovery of a novel feedback loop in which the PPM1A protein is significantly upregulated after TGF-β treatment. Feedback loops have crucial importance in dynamical systems because they create nonlinear responses and permit self-regulation (by converting a directed subgraph into a connected subgraph). In our study, the new feedback loop via PPM1A was significant enough to allow the model to finally explain the observed trends of phospho-R-Smad decline after TGF-β treatment (Figure 6, yellow versus green curves). Because PTEN is known to stabilize PPM1A against degradation [28], we built a model to illustrate hypothetical dynamics of PTEN-induced PPM1A sequestration, including delayed enzymatic activity for PPM1A. Note that in previous experiments, the influence of PTEN served as a positive feedback loop (PTEN-induced stabilization was inhibited by TGF-β [28]), not negative feedback. HaCaT cells are an accepted model system for understanding how epithelial cells respond to TGF-β; and it will be interesting for future work to test which cell types utilize PPM1A regulation for negative feedback.

Model 8 shows that PPM1A Stabilization, with delayed nuclear import, was sufficient to reconcile the early upregulation of PPM1A total protein with later decline of phospho-R-Smad. Our theoretical model could be useful for the design of experiments to determine how the upregulation actually occurs. Future work should test whether PTEN stabilizes and/or sequesters PPM1A in HaCaT after TGF-β treatment, as illustrated in Model 8. Our model would recommend testing for PPM1A-PTEN binding at 30 min–1 hr to catch the peak interaction, but testing for increased PPM1A activity at 4 hr, significantly later than the upregulation.

Careful examination of a broader set of previous work reveals some issues that appear to be discrepancies. The steepness of phospho-R-Smad decline in HaCaT appears to differ slightly between the experiments of Massague and colleagues in [11], [21] versus the experiments of Lin *et al*. [9], which are similar to our results (Figure 4C) and similar to the results of [12]. One possible explanation is a difference in the effective concentrations of TGF-β. TGF-β has a very short half-life, and the dissolving conditions, such as carrier protein concentration, can alter the effective concentration of TGF-β. Previous authors did not report how their TGF-β was dissolved, but we found that dissolving TGF-β without carrier protein led to a steeper decline of phospho-R-Smad, similar to Massague *et al*. [11], [21] (data not shown). We believe this slight discrepancy in slope is a technicality of the experiments and not fundamental to the pathway analysis.

Recent work has shown the importance of TGF-β depletion as a determinant of Smad signaling kinetics, for cells treated with low doses of TGF-β (10pM and 25pM) [47]. Our work did not emphasize low-dose contexts, but our models are consistent with observed TGF-β depletion behaviors. Figure S6 (Text S6) shows simulations of our final model, Model 8 except with lower doses of TGF-β treatment. Smad signaling was indeed dominated by TGF-β scarcity. When the Smad system was externally limited by TGF-β availability, self-limiting mechanisms and negative regulatory effects were not apparent. Negative self-regulation of the Smad system was strongly apparent in treatments with 2 ng/ml (80pM) of TGF-β, which is the dose studied in most previous experimental and computational studies of Smad dynamics.

After successfully predicting PPM1A upregulation and achieving recapitulation of the available datasets, our final contribution was to address an existing controversy about the role of proteasomal degradation in Smad signaling. We discovered that an apparent conflict about the role of degradation was in fact a mutually consistent set of trajectories that can both emerge from a single model. Degradation is intuitively understood to be a cumulative effect seen in long-term observations, but in this case the duration of observation was irrelevant, and the crucial variable for degradation was the duration of the TGF-β stimulus. MG132 (an inhibitor of proteasomal degradation) caused negligible change in pSmad2 levels (at 1,2,4,6 hr), in a system triggered with 30 min exposure to TGF-β, but MG132 caused a significant change in pSmad2 levels (at 1,2,4,6 hr), in a system triggered with long exposure to TGF-β. In other words, the importance of degradation in Smad signaling depended not on the time point at which pSmad2 was measured, but rather on the duration with which the Smad system had been induced. The consistency between the two experiments can be rationalized in retrospect because degradation depends on the area under the curve, which is large in systems with prolonged stimulus, and very small in systems with short stimulus. However, the consistency between Lin *et al*. and Massague *et al*. was not apparent prior to modeling, and mathematical inference of kinetic implications is dramatically different from the interpretations provided by the previous authors.

Computational modeling of any biochemical pathway involves several caveats and approximations, particularly when the system is as complex as Smad signaling. For our modeling of the Smad system, many interaction partners and post-translational modifications have been neglected, and some highly complex processes have been described as two-species reactions with simple mass action kinetics. Few of the rate constants have been determined from direct experiments and therefore, many parameters have been estimated by optimizing the fit between the model and the available datasets. Despite these limitations, we believe mathematical modeling provides valuable insights.

Our modeling provides a consistent, quantitative, and fine-grained integration of available information about the negative regulation of phospho-R-Smad, both from published literature and from our experiments. Our combination of modeling and experiments showed that previous negative regulatory effects such as Receptor Degradation have a minor effect, and led us to introduce a negative feedback loop with upregulation of PPM1A. Modeling can make additional predictions (e.g., future experiments should test for peak perturbation of PPM1A binding and activity). Also, modeling has provided a new and non-obvious interpretation for the effects of MG132 treatment. When interpreting the biological meaning of observed kinetics, informal intuition can unwittingly lead to flawed conclusions. Our modeling of Smad signaling may in the future be useful to other researchers interpreting data, designing experiments, or strategizing therapeutic perturbations.

## Materials and Methods

### Model Specifications

#### 1. Model structures

The reactions in our TGF-β signaling pathway model can be grouped into three sections: trafficking, Smad nucleocytoplasmic shuttling, and negative regulatory effects. Our assumptions of the receptor trafficking followed those in [25]. For Smad nucleocytoplasmic shuttling, we followed [10]. The only difference in Smad nucleocytoplasmic shuttling between [25] and [10] is that in [10], R-Smad can form a homogeneric complex. It has been shown that R-Smad can form complex with themselves [47]. Although the stoichiometry is not clear, we follow the simplest assumption in [10] that R-Smad can form homogeneric and heterogeneric complexes at the same rate. For the negative regulatory effects, we tested many possibilities based on literature findings and also our hypotheses (such as PPM1A Stabilization). Different effects are listed in Table 1. Here we describe each of them in details. All species names are listed in Table S1. All rate constants are listed in Table S2.

1. R-Smad Dephosphorylation was modeled as a single reaction in which nuclear phospho-R-Smad was dephosphorylated to R-Smad. The rate of dephosphorylation was proportional to the concentration of phospho-R-Smad (Reaction 32, Table S3).
2. Receptor Degradation was modeled by allowing the ligand-receptor complex in the caveolae to degrade at a rate proportional to the concentration of Smad7 (Reaction 31, Table S3).
3. P-Smad Degradation was modeled as a single reaction in which nuclear phospho-R-Smad was degraded at a rate proportional to its concentration (Reaction 33, Table S3), assuming that Smurf2 would be unchanged in TGF-β signaling.
4. Endogenous Synthesis and Degradation of R-Smad was modeled by incorporating (a) constant production of cytoplasmic R-Smad (Reaction 34, Table S3) and (b) degradation of R-Smad (including phosphorylated and unphosphorylated forms, excluding Smad complexes), proportional to the R-Smad concentration (Reactions 35-38, Table S3), but independent of Smurf2-induced degradation.
5. Receptor Inhibition was modeled (in Model S1) such that I-Smad could induce degradation, inhibition, and dephosphorylation of the ligand-receptor complex. Firstly, I-Smad (Smad7) was produced at a rate proportional to the concentration of Smad complex in the nucleus (Reactions 39-40, Table S3) and had a turnover rate proportional to its concentration (Reaction 41, Table S3). Then I-Smad could either associate with ligand receptor complex (LRC) in the caveolae (LRC_Cave_, Reaction 43, Table S3) or could associate with LRC in the early endosome (LRC_EE_, Reaction 44, Table S3). After association of the LRC with I-Smad, the complex could either be dephosphorylated (Reactions 45-46, Table S3) or degraded (Reactions 47-48, Table S3).
6. PPM1A Upregulation by Expression (in Model S2) assumed that the Smad complex in the nucleus was responsible for inducing PPM1A production. That is, the rate of induced production was proportional to the concentration of Smad complex in the nucleus (Reactions 55–56, Table S3). To simulate basal (unstimulated) levels, PPM1A was also synthesized at a constant rate (Reaction 51, Table S3). All sources of PPM1A, unless bound, were degraded endogenously at a rate proportional to PPM1A concentration (Reaction 51, Table S3). Another assumption concerns the kinetics of PPM1A activity. Prior models with constant PPM1A levels used a one-step approximation for the kinetics of the dephosphorylation of phospho-R-Smad by PPM1A, but the models with explicit regulation of PPM1A employed a two-step model of catalysis (Reaction 57–60, Table S3) with reversible association/dissociation followed by irreversible catalysis.
7. PPM1A Stabilization assumed that PTEN could associate with phospho-R-Smad (Reaction 64, Table S3) and this binary complex could further associate with PPM1A to form a ternary complex pSmad2:PTEN:PPM1A (Reaction 66, Table S3). The ternary complex could dissociate in the manner it was formed, or could alternatively release the phospho-R-Smad alone and the PTEN-PPM1A as a binary complex (Reaction 67, Table S3). The PTEN-PPM1A complex was assumed to evade degradation while the unbound PPM1A would degrade spontaneously (Reactions 52–53, Table S3). PPM1A was synthesized in the cytoplasm (Reaction 52, Table S3) and was imported into the nucleus at a high rate (Reaction 54, Table S3) so that PPM1A was predominantly in the nucleus. When PTEN bound to PPM1A, PPM1A phosphatase activity was assumed to be unchanged (Reaction 61–64, Table S3). We allowed the rate of PTEN-PPM1A imported into the nucleus to differ from the rate of import for unbound PPM1A, and the actual rates were estimated numerically (Reaction 70, Table S3). PTEN in the nucleus could be exported back into the cytoplasm (Reaction 71, Table S3).

#### 2. Kinetic parameters

*2.1 Rate constants*. The list of rate constants is shown in Table S2. We have retained the values of the experimentally derived parameters cited by [25]. We have also retained previous rates for the type I and the type II receptors and the recycling rate of the ligand-receptor complex in the caveolae, which had been strongly constrained by qualitative information. For the other rate constants that were estimated by [25], we have re-estimated these parameters again in the context of our model. In particular, the rate constants for Smad nucleocytoplasmic shuttling were modified to fit our model calibration and the new findings in [10]. First of all, the concentrations of species in the nucleus are represented as their relative concentrations in the cytoplasm. For example, if the absolute concentration of Smad2 in the nucleus is, we use the relative concentration instead of in our model to simulate the concentrations in two compartments. For example, if the absolute concentration of Smad2 in the nucleus is 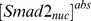, we use the relative concentration 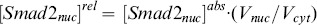 instead of 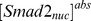 in our model to simulate the concentrations in two compartments. For example, the ODEs of Smad2 using absolute concentrations are:
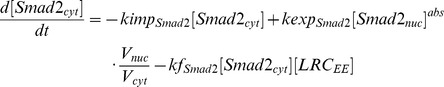


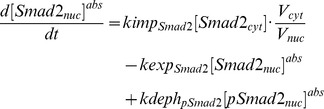
After substituting 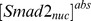 with 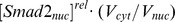:
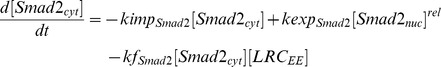


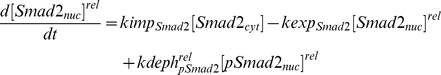
where
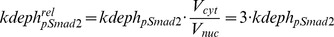



The export rates computed in [6] are identical to our 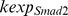
and 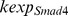. However, the import in [6] are actually 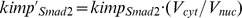 and 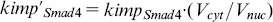. So our import rates are 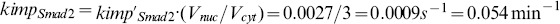 and 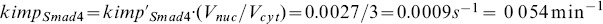. Note that the rates of the reactions in the nucleus (e.g. 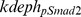) are substituted because the concentrations of the reactants are relative. But all these rates are estimated so we do not need to substitute any values of them.

Secondly, it was found that the import rate of Smad complex is higher than the monomeric Smad2 [10]. Therefore, we set the import and export rate of Smad complex according to [10].

*2.2 Initial concentrations*. The initial concentrations of the receptors followed those in [25], as the rate constants for receptor trafficking were kept the same as those in [25]. For Smad2 and Smad4, we can derive their equilibrium concentrations based on their shuttling rates and total amount. At steady state,
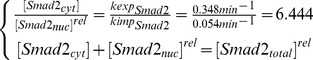


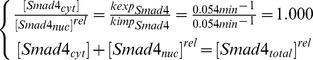
The relative concentrations of total Smad2 and total Smad4 in HaCaT cells are 571.43 nM and 1333.33 nM [12], [25].

For each model, all initial concentrations were set to their equilibrium points (i.e., their steady-state levels) in unstimulated cells, computed by simulating the system for 10^4^ minutes (1 week) without TGF-β. The initial concentrations are shown in Table S1.

Note that in the absence of TGF-β stimulation, only a small number of reactions occur, mainly production, degradation, and trafficking. The rate parameters for these reactions were the same for all models (Tables S2 and S5), causing all models to have the same steady-state concentrations, for all species they share in common. Note that some species are specific to certain models, such as cytosolic PPM1A appearing in (Model 8 and S3). This modification does not affect the steady-state levels of the other species, because the reactions catalyzed by PPM1A do not occur until after TGF-β stimulation.

*2.3 Model simulation and parameter estimation*. The model simulation and parameter estimation were performed using KroneckerBio toolbox in Matlab. The KroneckerBio toolbox basically calls the ode15 s function in Matlab to solve the system of ODE equations and the fmincon function in Matlab to estimate parameters. Multiple initial guesses were generated randomly in order to achieve a more global optimum in parameter estimations. Sum of squared errors were used as the objective function to optimize the model to experimental data.

The parameters related to each negative regulatory effect are listed in Table S4. The best-fit parameters of each model are listed in Table S5.

### Biological Assays

#### 1. HaCaT cell culture and TGF-β treatment

HaCaT cells (from Cell Lines Service) were cultured following the protocol provided by the manufacturer. DMEM culture medium with 10% FBS was used to culture the cells. DMEM culture medium without FBS was used during treatment of TGF-β.

#### 2. ELISA for phosphorylated Smad2 and total Smad2

ELISA kits (from Cell Signaling) were used to quantify phosphorylated Smad2 and total Smad2. Whole cell lysates were collected using attached cell lysis buffer and following the cell lysis protocol in the kits. Sample dilutions for phosphorylated Smad2 and total Smad2 are 1 time and 100 times respectively. Serial dilutions of one sample were measured to check the linear range of the readouts.

#### 3. Western blot for total type I receptor and PPM1A

Whole cell lysates were collected using RIPA buffer from HaCaT cells for western blots. Antibodies against the type I receptor (Santa Cruz) and PPM1A (Abcam) were used following manufacturers' supplier's instructions. Primary antibody dilutions of 1∶7500 and 1∶250 were used for the type I receptor and PPM1A respectively. The quantification of the band intensities was preformed using ImageJ.

## Supporting Information

Figure S1 Analysis of receptor internalization in Model 1. (**A**) Perturbation analysis of the rate of ligand-receptor complex (LRC) internalization. Log Parameter Perturbation is the log ratio of perturbed LRC internalization rate (the rate for internalizing early endosome and caveolae were changed with the same ratio) to its original value. Blue curve shows the result of perturbation analysis in Model 1, while red curve shows the result of perturbation analysis after changing the production rates of T1R (10-fold decrease) and T2R (10-fold increase) in Model 1. (**B**) Inhibition of LRC internalization in Model 1 after changing the production rates of T1R (10-fold decrease) and T2R (10-fold increase). Curves change from blue to red as LRC internalization rate decreases from 1 to 10^−3^ in log scale. (**C**) Adaptation Index change (x-axis) with 10-fold increase of each parameter. (**D**) Adaptation Index change (x-axis) with 10-fold decrease of each parameter. (**E**) Dose response at 45 min. (**F**) Dose response at 45 min. In panel E and F, blue curve shows dose response in Model 1, while red curve shows dose response in Model 1 after changing the production rates of T1R (10-fold decrease) and T2R (10-fld increase) in Model 1.
(EPS)

Figure S2 Fitted models with extended receptor inhibition effects (Model S1). Each blue point represents a single model. The x-axis is the type I receptor (T1R) level simulated at 24 hr. The y-axis is the ratio of I-Smad-bound ligand-receptor complex in the early endosome and caveolae. We rescaled the axes to better visualize the majorities of the data points (upper left sub-figure). Models in the red box region should be able to explain both the type I receptor level and localization of I-Smad. However, no fitted model falls in the red box.
(EPS)

Figure S3 Sensitivity analysis heat map of Model 5. (**A**) The sensitivity of the P-Smad degradation rate to each species with relative perturbations of the rate from 10^−4^ to 10^2^. (**B**) The sensitivity of the receptor degradation rate to each species with relative perturbations of the rate from 10^−4^ to 10^2^.
(EPS)

Figure S4 Model predictions PPM1A upregulation. (**A**) Model S2, in which PPM1A is upregulated by Smad complex in the nucleus, was fitted to the long-exposure and the short-exposure phospho-R-Smad experimental data. (**B**) Model S2 predicted PPM1A upregulation under long-exposure of TGF-β (green curve). (**C**) Model S2 predicted unchanged T1R levels (green curve), in agreement with our experimental results (green dots). (**D**) Model S2 predicted unchanged total R-Smad levels (green curve), in agreement with our experimental results (green dots).
(EPS)

Figure S5 Model-based predictions of PPM1A Stabilization dynamics. (**A**) Model S3, in which PPM1A is stabilized by PTEN, was fitted to the long-exposure and the short-exposure phospho-R-Smad experimental data. (**B**) Model S3 predicted early PPM1A upregulation (within 1hr) under long-exposure treatments with TGF-β (green curve). This is in agreement with our experimental measurements of PPM1A (greed dots). (**C**) Model S3 predicted unchanged T1R levels (green curve), in agreement with our experimental results (green dots). (**D**) Model S3 predicted unchanged total R-Smad levels (green curve), in agreement with our experimental results (green dots).
(EPS)

Figure S6 TGF-β dose response. (**A**) Simulated P-Smad2 levels at 45 min under different doses of TGF-β treatment. (**B**) Simulated P-Smad2 levels at 24 hr under different doses of TGF-β treatment. (**C**) Simulations of the P-Smad2 dynamics with different doses of TGF-β. The color of the curve turns from blue to red as TGF-β dose increases (0.025, 0.0625, 0.125, 0.25, 0.5, 1, 2 ng/ml).
(EPS)

Figure S7 Sum of Squared Errors (SSE) of the best-fit models. SSE was plotted separately for P-Smad2 and PPM1A in Model 8, S2 and S3 so that the goodness-of-fit of these models can be compared to other models.
(EPS)

Figure S8 Modeling cycloheximide treatment. (**A**) Dataset originated from [49] and quantified from [50]. The blue dots show P-Smad2 levels in the nucleus in HaCaT cells after treated with 2 ng/ml of TGF-β. The red dots show P-Smad2 levels under the same condition but HaCaT cells were pretreated with cycloheximide for 30 min. (**B**) Simulated P-Smad2 levels from Model 8 without cycloheximide treatment (blue curve) and with cycloheximide treatment (all production rates are half of their original values, red curve).
(EPS)

Figure S9 The dynamics of the PTEN-PPM1A complex in Model 8. In this model, we assumed PTEN is initially cytoplasmic, and we assumed that phospho-R-Smad could induce association between PTEN and PPM1A. The simulation shows that TGF-β stimulation would rapidly induce the formation of a cytosolic PTEN-PPM1A complex (blue curve), which would then translocate to the nucleus. The speed of nuclear translocation would affect the functional availability of PPM1A toward phospho-R-Smad in the nucleus. If PTEN binding slows the nuclear translocation of PPM1A (as simulated in Model 8), then PTEN-induced stabilization could have a complex effect of causing a short-term delay in PPM1A availability even though it causes a long-term increase in PPM1A abundance. Note that TGF-β does not alter the total expression of PTEN (green curve) in this model.
(EPS)

Table S1 Initial Concentrations (I.C.) in nM.
(PDF)

Table S2 Rate constants.
(PDF)

Table S3 Reactions table: All reactions in Models1–8 and Models S1–S3 with rate constants labeled.
(PDF)

Table S4 Table of negative regulatory effects and their related rate constants.
(PDF)

Table S5 Table of estimated parameters in Models 1–8 and Models S2–S3.
(PDF)

Text S1 The rate of receptor internalization does not affect the peak and decline of phospho-R-Smad when TGF-β is saturating.
(PDF)

Text S2 Extended I-Smad-mediated receptor inhibition.
(PDF)

Text S3 Sensitivity analysis.
(PDF)

Text S4 PPM1A upregulation could possibly be a slow-mode effects.
(PDF)

Text S5 Model-based prediction of PPM1A Stabilization dynamics.
(PDF)

Text S6 Transient and sustained signaling in the TGF-β signaling pathway.
(PDF)

Text S7 Evaluation of best-fits of all models.
(PDF)

Text S8 Modeling cycloheximide treatment.
(PDF)

Dataset S1 SBML file for Model 1.
(XML)

Dataset S2 SBML file for Model 2.
(XML)

Dataset S3 SBML file for Model 3.
(XML)

Dataset S4 SBML file for Model 4.
(XML)

Dataset S5 SBML file for Model 5.
(XML)

Dataset S6 SBML file for Model 6.
(XML)

Dataset S7 SBML file for Model 7.
(XML)

Dataset S8 SBML file for Model 8.
(XML)

Dataset S9 SBML file for Model S2.
(XML)

Dataset S10 SBML file for Model S3.
(XML)
