# Correction: The Self-Limiting Dynamics of TGF-β Signaling *In Silico* and *In Vitro*, with Negative Feedback through PPM1A Upregulation

**DOI:** 10.1371/journal.pcbi.1003832

**Published:** 2014-08-08

**Authors:** The PLOS Computational Biology Staff

There are affiliations missing for the corresponding author. The full affiliation for Hanry Yu should read:

Institute of Bioengineering and Nanotechnology, Agency for Science, Technology and Research, Singapore, Computational and Systems Biology, Singapore-MIT Alliance, Singapore, BioSyM, Singapore-MIT Alliance for Research and Technology, Singapore, Mechanobiology Institute, Singapore, NUS Graduate School for Integrative Science and Engineering, National University of Singapore, Singapore, Department of Physiology, National University of Singapore, Singapore, Department of Biological Engineering, Massachusetts Institute of Technology, Cambridge, Massachusetts, United States of America.
