# Supplementary material for: The Self-Limiting Dynamics of TGF-β Signaling In Silico and In Vitro, with Negative Feedback through PPM1A Upregulation
Source: PLoS Comput Biol. 2014 Jun 5;10(6):e1003573. doi: 10.1371/journal.pcbi.1003573 (PMC4105941; doi:10.1371/journal.pcbi.1003573)
Supplement: Table S1 — Initial Concentrations (I.C.) in nM. (PDF) [file pcbi.1003573.s010.pdf]

**Table S1**: Initial Concentrations (I.C.) in nM.

| Species Name | Species Description | I.C. | Ref. | Model |
|---|---|---|---|---|
| TGF-β | The TGF-β ligand | 80 | [1] | All |
| $T1R_{Surf}$ | The type I receptor on the cell surface | 0.237 | [1] | All |
| $T1R_{Cave}$ | The type I receptor in the caveolae | 2.092 | [1] | All |
| $T1R_{EE}$ | The type I receptor in the early endosome | 2.06 | [1] | All |
| $T2R_{Surf}$ | The type II receptor on the cell surface | 0.202 | [1] | All |
| $T2R_{Cave}$ | The type II receptor in the caveolae | 1.778 | [1] | All |
| $T2R_{EE}$ | The type II receptor in the early endosome | 1.148 | [1] | All |
| $pT2R_{Surf}$ | Ligand-bound type II receptor on the cell surface | 0 | | All |
| $pT2R_{Cave}$ | Ligand-bound type II receptor in the caveolae | 0 | | All |
| $pT2R_{EE}$ | Ligand-bound type II receptor in the early endosome | 0 | | All |
| $LRC_{Surf}$ | The ligand-receptor complex on the cell surface | 0 | | All |
| $LRC_{Cave}$ | The ligand-receptor complex in the caveolae | 0 | | All |
| $LRC_{EE}$ | The ligand-receptor complex in the early endosome | 0 | | All |
| T1R:T2R | The T1R-T2R complex released from LRC | 0 | | All |
| RI | The receptor inhibitor SB-431542 | 600 | [2] | All |
| $LRC_{EE}$:RI | The complex of the receptor inhibitor and LRC | 0 | | All |
| LRC:Smad2 | The complex of Smad2 and LRC | 0 | | All |
| $Smad2_{Cyt}$ | Smad2 in the cytoplasm | 494.67 | [1] | All |
| $Smad2_{Nuc}$ | Smad2 in the nucleus | 76.76 | [1] | All |
| $pSmad2_{Cyt}$ | Phosphorylated Smad2 in the cytoplasm | 0 | | All |
| $pSmad2_{Nuc}$ | Phosphorylated Smad2 in the nucleus | 0 | | All |
| $Smad4_{Cyt}$ | Smad4 in the cytoplasm | 666.65 | [1] | All |
| $Smad4_{Nuc}$ | Smad4 in the nucleus | 666.65 | [1] | All |
| Smad2:$Smad4_{Cyt}$ | The complex of Smad2 and Smad4 in the cytoplasm | 0 | | All |
| Smad2:$Smad4_{Nuc}$ | The complex of Smad2 and Smad4 in the nucleus | 0 | | All |
| Smad2:$Smad2_{Cyt}$ | The homo-dimer of Smad2 in the cytoplasm | 0 | | All |
| Smad2:$Smad2_{Nuc}$ | The homo-dimer of Smad2 in the nucleus | 0 | | All |
| Smad7 | Smad7 | 100 | | 2,3,5,S1 |
| $dephLRC_{Cave}$ | Dephosphorylated LRC in the caveolae | 0 | | S1 |
| $dephLRC_{EE}$ | Dephosphorylated LRC in the early endosome | 0 | | S1 |
| $LRC_{Cave}$:Smad7 | The complex of Smad7 and LRC in the caveolae | 0 | | S1 |
| $LRC_{EE}$:Smad7 | The complex of Smad7 and LRC in the early endosome | 0 | | S1 |
| PPM1A | Overall PPM1A | 10 | | S2 |
| $PPM1A_{Cyt}$ | PPM1A in the cytoplasm | 50 | | 8,S3 |
| $PPM1A_{Nuc}$ | PPM1A in the nucleus | 50 | | 8,S3 |
| $PTEN_{Cyt}$ | PTEN in the cytoplasm | Estimated | | 8,S3 |
| $PTEN_{Nuc}$ | PTEN in the nucleus | 0 | | 8,S3 |
| pSmad2:$PPM1A_{Cyt}$ | The complex of pSmad2 and PPM1A in the cytoplasm | 0 | | 8,S3 |
| pSmad2:PPM1A:$PTEN_{Cyt}$ | The complex of pSmad2 and PPM1A:PTEN in the cytoplasm | 0 | | 8,S3 |
| pSmad2:$PTEN_{Cyt}$ | The complex of pSmad2 and PTEN in the cytoplasm | 0 | | 8,S3 |
| pSmad2:PTEN:$PPM1A_{Cyt}$ | The complex of pSmad2:PTEN and PPM1A in the cytoplasm | 0 | | 8,S3 |
| PPM1A:$PTEN_{Cyt}$ | The PPM1A-PTEN complex released from pSmad2:PTEN:PPM1A in the cytoplasm | 0 | | 8,S3 |
| pSmad2:$PPM1A_{Nuc}$ | The complex of pSmad2 and PPM1A in the nucleus | 0 | | 8,S2,S3 |
| pSmad2:PPM1A:$PTEN_{Nuc}$ | The complex of pSmad2 and PPM1A:PTEN in the nucleus | 0 | | 8,S3 |
| PPM1A:$PTEN_{Nuc}$ | The PPM1A-PTEN complex released from pSmad2:PTEN:PPM1A in the nucleus | 0 | | 8,S3 |

*Nuclear species have been expressed relative to the concentrations in cytoplasm.*

*1. Klipp E, Zi Z (2007) Constraint-based modeling and kinetic analysis of the smad dependent tgf-Beta signaling pathway. PLoS ONE 2: e936.*

*2. Schmierer B, Tournier AL, Bates PA, Hill CS (2008) Mathematical modeling identifies Smad nucleocytoplasmic shuttling as a dynamic signal-interpreting system. Proc Natl Acad Sci U S A 105: 6608-6613.*
