# Supplementary material for: The Self-Limiting Dynamics of TGF-β Signaling In Silico and In Vitro, with Negative Feedback through PPM1A Upregulation
Source: PLoS Comput Biol. 2014 Jun 5;10(6):e1003573. doi: 10.1371/journal.pcbi.1003573 (PMC4105941; doi:10.1371/journal.pcbi.1003573)
Supplement: Table S2 — Rate constants. (PDF) [file pcbi.1003573.s011.pdf]

Table S2: Rate Constants

Rate Constant Name	Values	Reference
v_{T1R}	0.0103	(Klipp 2007)
v_{T2R}	0.02869	(Klipp 2007)
ki_{EE}	0.33	(Klipp 2007)
kr_{EE}	0.033	(Klipp 2007)
ki_{Cave}	0.33	(Klipp 2007)
kr_{Cave}	0.03742	(Klipp 2007)
$kdeg_{T1R}$	0.005	(Klipp 2007)
$kdeg_{T2R}$	0.025	(Klipp 2007)
kcd	0.005	(Klipp 2007)
$kLRC_1$	estimated	
$kLRC_2$	estimated	
$kr_{Receptor}$	10000	
kfSmad2	estimated	
krSmad2	estimated	
kfSmadsComplex	estimated	
krSmadsComplex	estimated	
$kimp_{Smad2}$	0.054	(Schmierer 2008)
$kexp_{Smad2}$	0.348	(Schmierer 2008)
$kimp_{Smad4}$	0.054	(Schmierer 2008)
$kexp_{Smad4}$	0.054	(Schmierer 2008)
$kimp_{SmadsComplex}$	0.27	(Schmierer 2008)
klid	estimated	
$kdeph_{pSmad2}$	estimated	
$kdeg_{pSmad2}$	estimated	
v_{Smad2}	estimated	
$kdeg_{Smad2}$	estimated	
kSmad7	estimated	
$kfSmad7_{Cave}$	estimated	
$kbSmad7_{Cave}$	estimated	
$kfSmad7_{EE}$	estimated	
$kbSmad7_{EE}$	estimated	
$kdeph_{LRC:Smad7}$	estimated	
$kdeg_{LRC:Smad7}$	estimated	
v_{PPM1A}	estimated	
$kdeg_{PPM1A}$	estimated	
kfPPM1A	estimated	
kbPPM1A	estimated	
krPPM1A	estimated	
$kdeph_{PPM1A}$	estimated	
kfPP	estimated	
kbPP	estimated	
krPP	estimated	
kfPTEN	estimated	
kbPTEN	estimated	
$kimp_{PPM1A}$	estimated	
$kimp_{PP}$	estimated	
$kexp_{PP}$	estimated	
$kexp_{PTEN}$	estimated	

kfRI	100	(Schmierer 2008)
kbRI	684	(Schmierer 2008)
