# Supplementary material for: The Self-Limiting Dynamics of TGF-β Signaling In Silico and In Vitro, with Negative Feedback through PPM1A Upregulation
Source: PLoS Comput Biol. 2014 Jun 5;10(6):e1003573. doi: 10.1371/journal.pcbi.1003573 (PMC4105941; doi:10.1371/journal.pcbi.1003573)
Supplement: Table S3 — Reactions table: All reactions in Models1–8 and Models S1–S3 with rate constants labeled. (PDF) [file pcbi.1003573.s012.pdf]

Table S3: Reactions Table: All reactions in Models1-8 and Models S1-S3 with rate constants labeled.

	Reaction	Models
1	$\xrightarrow{v_{T1R}} T1R_{Surf}$	All
2	$\xrightarrow{v_{T2R}} T2R_{Surf}$	All
3	$T1R_{Surf} \underset{kr_{EE}}{\overset{ki_{EE}}{\rightleftharpoons}} T1R_{EE}$	All
4	$T1R_{Surf} \underset{kr_{Cave}}{\overset{ki_{Cave}}{\rightleftharpoons}} T1R_{Cave}$	All
5	$T2R_{Surf} \underset{kr_{EE}}{\overset{ki_{EE}}{\rightleftharpoons}} T2R_{EE}$	All
6	$T2R_{Surf} \underset{kr_{Cave}}{\overset{ki_{Cave}}{\rightleftharpoons}} T2R_{Cave}$	All
7	$pT2R_{Surf} \underset{kr_{EE}}{\overset{ki_{EE}}{\rightleftharpoons}} pT2R_{EE}$	All
8	$pT2R_{Surf} \underset{kr_{Cave}}{\overset{ki_{Cave}}{\rightleftharpoons}} pT2R_{Cave}$	All
9	$T1R_{EE} \xrightarrow{kdeg_{T1R}}$	All
10	$T2R_{EE} \xrightarrow{kdeg_{T2R}}$	All
11	$pT2R_{EE} \xrightarrow{kdeg_{T2R}}$	All
12	$TGF\beta + T2R_{Surf} \xrightarrow{kLRC_1} pT2R_{Surf}$	All
13	$pT2R_{Surf} + T1R_{Surf} \xrightarrow{kLRC_2} LRC_{Surf}$	All
14	$LRC_{Surf} \xrightarrow{ki_{EE}} LRC_{EE}$	All
15	$LRC_{EE} \xrightarrow{kr_{EE}} T1R:T2R + TGF\beta$	All
16	$LRC_{Surf} \xrightarrow{ki_{Cave}} LRC_{Cave}$	All
17	$LRC_{Cave} \xrightarrow{kr_{Cave}} T1R:T2R + TGF\beta$	All
18	$T1R:T2R \xrightarrow{krR} T1R_{Surf} + T2R_{Surf}$	All
19	$LRC_{EE} \xrightarrow{kcd}$	All

	Reaction	Models
20	$LRC_{EE} + Smad2_{Cyt} \xrightarrow{kfSmad2} LRC : Smad2$	All
21	$LRC : Smad2 \xrightarrow{krSmad2} LRC_{EE} + pSmad2_{Cyt}$	All
22	$pSmad2_{Cyt} + Smad4_{Cyt} \xrightarrow{kfSmadsComplex} Smad2 : Smad4_{Cyt}$	All
23	$pSmad2_{Cyt} + pSmad2_{Cyt} \xrightarrow{kfSmadsComplex} Smad2 : Smad2_{Cyt}$	All
24	$pSmad2_{Nuc} + Smad4_{Nuc} \underset{krSmadsComplex}{\overset{kfSmadsComplex}{\rightleftharpoons}} Smad2 : Smad4_{Nuc}$	All
25	$pSmad2_{Nuc} + pSmad2_{Nuc} \underset{krSmadsComplex}{\overset{kfSmadsComplex}{\rightleftharpoons}} Smad2 : Smad2_{Nuc}$	All
26	$Smad2_{Cyt} \underset{kexp_{Smad2}}{\overset{kimp_{Smad2}}{\rightleftharpoons}} Smad2_{Nuc}$	All
27	$pSmad2_{Cyt} \underset{kexp_{Smad2}}{\overset{kimp_{Smad2}}{\rightleftharpoons}} pSmad2_{Nuc}$	All
28	$Smad4_{Cyt} \underset{kexp_{Smad4}}{\overset{kimp_{Smad4}}{\rightleftharpoons}} Smad4_{Nuc}$	All
29	$Smad2 : Smad4_{Cyt} \xrightarrow{kimp_{SmadsComplex}} Smad2 : Smad4_{Nuc}$	All
30	$Smad2 : Smad2_{Cyt} \xrightarrow{kimp_{SmadsComplex}} Smad2 : Smad2_{Nuc}$	All
31	$LRC_{Cave} + Smad7 \xrightarrow{klid} Smad7$	Model(2,3,5)
32	$pSmad2_{Nuc} \xrightarrow{kdeph_{pSmad2}} Smad2_{Nuc}$	Model(1,2,5,6,7,S1)
33	$pSmad2_{Nuc} \xrightarrow{kdeg_{pSmad2}}$	Model(4,5,6,7,8)
34	$\xrightarrow{v_{Smad2}} Smad2_{Cyt}$	Model(7,8)
35	$Smad2_{Cyt} \xrightarrow{kdeg_{Smad2}}$	Model(7,8)
36	$Smad2_{Nuc} \xrightarrow{kdeg_{Smad2}}$	Model(7,8)
37	$pSmad2_{Cyt} \xrightarrow{kdeg_{Smad2}}$	Model(7,8)
38	$pSmad2_{Nuc} \xrightarrow{kdeg_{Smad2}}$	Model(7,8)
39	$Smad2 : Smad4_{Nuc} \xrightarrow{kSmad7} Smad2 : Smad4_{Nuc} + Smad7$	Model(S1)

	Reaction	Models
40	$Smad2:Smad2_{Nuc} \xrightarrow{kSmad7} Smad2:Smad2_{Nuc} + Smad7$	Model(S1)
41	$Smad7 \xrightarrow{kcd}$	Model(S1)
42	$LRC_{EE}:Smad7 \xrightarrow{kcd}$	Model(S1)
43	$LRC_{Cave} + Smad7 \underset{krSmad7_{Cave}}{\overset{kfSmad7_{Cave}}{\rightleftharpoons}} LRC_{Cave}:Smad7$	Model(S1)
44	$LRC_{EE} + Smad7 \underset{krSmad7_{EE}}{\overset{kfSmad7_{EE}}{\rightleftharpoons}} LRC_{EE}:Smad7$	Model(S1)
45	$LRC_{Cave}:Smad7 \xrightarrow{kdeph_{LRC:Smad7}} dephLRC_{Cave} + Smad7$	Model(S1)
46	$LRC_{EE}:Smad7 \xrightarrow{kdeph_{LRC:Smad7}} dephLRC_{EE} + Smad7$	Model(S1)
47	$LRC_{Cave}:Smad7 \xrightarrow{kdeg_{LRC:Smad7}}$	Model(S1)
48	$LRC_{EE}:Smad7 \xrightarrow{kdeg_{LRC:Smad7}}$	Model(S1)
49	$dephLRC_{Cave} \xrightarrow{kr_{Cave}} T1R:T2R + TGF\beta$	Model(S1)
50	$dephLRC_{EE} \xrightarrow{kr_{EE}} T1R:T2R + TGF\beta$	Model(S1)
51	$\underset{kdeg_{PPM1A}}{\overset{v_{PPM1A}}{\rightleftharpoons}} PPM1A_{Nuc}$	Model(S2)
52	$\underset{kdeg_{PPM1A}}{\overset{v_{PPM1A}}{\rightleftharpoons}} PPM1A_{Cyt}$	Model(8,S3)
53	$PPM1A_{Nuc} \xrightarrow{kdeg_{PPM1A}}$	Model(8,S3)
54	$PPM1A_{Cyt} \xrightarrow{kimp_{PPM1A}} PPM1A_{Nuc}$	Model(8,S3)
55	$Smad2:Smad4_{Nuc} \xrightarrow{kPPM1A} Smad2:Smad4_{Nuc} + PPM1A_{Nuc}$	Model(S2)
56	$Smad2:Smad2_{Nuc} \xrightarrow{kPPM1A} Smad2:Smad2_{Nuc} + PPM1A_{Nuc}$	Model(S2)
57	$pSmad2_{Nuc} + PPM1A_{Nuc} \underset{kbPPM1A}{\overset{kfPPM1A}{\rightleftharpoons}} pSmad2:PPM1A_{Nuc}$	Model(8,S2,S3)
58	$pSmad2:PPM1A_{Nuc} \xrightarrow{kdeph_{PPM1A}} Smad2_{Nuc} + PPM1A_{Nuc}$	Model(8,S2,S3)
59	$pSmad2_{Cyt} + PPM1A_{Cyt} \underset{kbPPM1A}{\overset{kfPPM1A}{\rightleftharpoons}} pSmad2:PPM1A_{Cyt}$	Model(8,S3)

	Reaction	Models
60	$pSmad2:PPM1A_{Cyt} \xrightarrow{kdeph_{PPM1A}} Smad2_{Cyt} + PPM1A_{Cyt}$	Model(8,S3)
61	$pSmad2_{Nuc} + PTEN:PPM1A_{Nuc} \underset{kbPPM1A}{\overset{kfPPM1A}{\rightleftharpoons}} pSmad2:PPM1A:PTEN_{Nuc}$	Model(8,S3)
62	$pSmad2:PTEN:PPM1A_{Nuc} \xrightarrow{kdeph_{PPM1A}} Smad2_{Nuc} + PTEN:PPM1A_{Nuc}$	Model(8,S3)
63	$pSmad2_{Cyt} + PTEN:PPM1A_{Cyt} \underset{kbPPM1A}{\overset{kfPPM1A}{\rightleftharpoons}} pSmad2:PPM1A:PTEN_{Cyt}$	Model(8,S3)
64	$pSmad2:PTEN:PPM1A_{Cyt} \xrightarrow{kdeph_{PPM1A}} Smad2_{Cyt} + PTEN:PPM1A_{Cyt}$	Model(8,S3)
65	$pSmad2_{Cyt} + PTEN_{Cyt} \underset{kbTEN}{\overset{kfPTEN}{\rightleftharpoons}} pSmad2:PTEN_{Cyt}$	Model(8,S3)
66	$pSmad2:PTEN_{Cyt} + PPM1A_{Cyt} \underset{kbPP}{\overset{kfPP}{\rightleftharpoons}} pSmad2:PTEN:PPM1A_{Cyt}$	Model(8,S3)
67	$pSmad2:PTEN:PPM1A_{Cyt} \xrightarrow{krPP} pSmad2_{Cyt} + PTEN:PPM1A_{Cyt}$	Model(8,S3)
68	$PTEN:PPM1A_{Cyt} \xrightarrow{krPPM1A} PTEN_{Cyt} + PPM1A_{Cyt}$	Model(8,S3)
69	$PTEN:PPM1A_{Nuc} \xrightarrow{krPPM1A} PTEN_{Nuc} + PPM1A_{Nuc}$	Model(8,S3)
70	$PTEN:PPM1A_{Cyt} \underset{kexp_{PP}}{\overset{kimp_{PP}}{\rightleftharpoons}} PTEN:PPM1A_{Nuc}$	Model(8,S3)
71	$PTEN_{Nuc} \xrightarrow{kexp_{PTEN}} PTEN_{Cyt}$	Model(8,S3)
72	$LRC_{EE} + RI \underset{kbRI}{\overset{kfRI}{\rightleftharpoons}} LRC_{EE}:RI$	All
