# Supplementary material for: The Self-Limiting Dynamics of TGF-β Signaling In Silico and In Vitro, with Negative Feedback through PPM1A Upregulation
Source: PLoS Comput Biol. 2014 Jun 5;10(6):e1003573. doi: 10.1371/journal.pcbi.1003573 (PMC4105941; doi:10.1371/journal.pcbi.1003573)
Supplement: Table S4 — Table of negative regulatory effects and their related rate constants. (PDF) [file pcbi.1003573.s013.pdf]

Table S4: Table of Negative Regulatory Effects and Their Related Rate Constants

	R-Smad Dephosphorylation	Receptor Degradation	P-Smad Degradation	Endogenous Synthesis and Degrdation of R-Smad	Receptor Inhibitions	PPM1A Upregulation by Expression	PPM1A Stabilization
$kdeph_{pSmad2}$	estimated	0	0	0	0	0	0
$kdeg_{pSmad2}$	0	0	estimated	estimated	0	0	0
v_{Smad2}	0	0	0	5.7143 $nMmin^{-1}$	0	0	0
$kdeg_{Smad2}$	0	0	0	0.01 min^{-1}	0	0	0
klid	0	estimated	0	0	0	0	0
kSmad7	0	0	0	0	estimated	0	0
$kfSmad7_{cave}$	0	0	0	0	estimated	0	0
$kbSmad7_{cave}$	0	0	0	0	estimated	0	0
$kfSmad7_{ee}$	0	0	0	0	estimated	0	0
$kbSmad7_{ee}$	0	0	0	0	estimated	0	0
$kdeph_{LRC}$	0	0	0	0	estimated	0	0
$kdeg_{LRC}$	0	0	0	0	estimated	0	0
v_{PPM1A}	0	0	0	0	0	0.1 $nMmin^{-1}$	7 $nMmin^{-1}$
$kdeg_{PPM1A}$	0	0	0	0	0	0.01 min^{-1}	0.07 min^{-1}
$kdeph_{PPM1A}$	0	0	0	0	0	estimated	estimated
kPPM1A	0	0	0	0	0	estimated	0
kfPPM1A	0	0	0	0	0	0	estimated
kbPPM1A	0	0	0	0	0	0	estimated
krPPM1A	0	0	0	0	0	0	estimated
kfPP	0	0	0	0	0	0	estimated
kbPP	0	0	0	0	0	0	estimated
kfPTEN	0	0	0	0	0	0	estimated
kbPTEN	0	0	0	0	0	0	estimated
$kimp_{PPM1A}$	0	0	0	0	0	0	0.07 min^{-1}
$kimp_{PP}$	0	0	0	0	0	0	estimated
$kexp_{PP}$	0	0	0	0	0	0	estimated
$kexp_{PTEN}$	0	0	0	0	0	0	estimated
