# Supplementary material for: The Self-Limiting Dynamics of TGF-β Signaling In Silico and In Vitro, with Negative Feedback through PPM1A Upregulation
Source: PLoS Comput Biol. 2014 Jun 5;10(6):e1003573. doi: 10.1371/journal.pcbi.1003573 (PMC4105941; doi:10.1371/journal.pcbi.1003573)
Supplement: Table S5 — Table of estimated parameters in Models 1–8 and Models S2–S3. (PDF) [file pcbi.1003573.s014.pdf]

Table S5: Table of Estimated Parameters in Models 1-8 and Models S2-S3.

	Model 1	Model 2	Model 3	Model 4	Model 5	Model 6	Model 7	Model 8	Model S2	Model S3
$kLRC_1$ ($nM^{-1}min^{-1}$)	213.3	79.09	1036	219.9	0.7669	2388	34.11	46.32	0.9068	2443
$kLRC_2$ ($nM^{-1}min^{-1}$)	3459	22.56	7900	62.49	4.677	0.1182	1025	0.04041	0.04956	0.0066
kfSmad2 ($nM^{-1}min^{-1}$)	0.03958	0.02861	8.385e-4	0.1599	0.04441	0.6500	0.01185	1.252	0.9100	6.930
krSmad2 ($nM^{-1}min^{-1}$)	2.443e5	123.6	8598	1332	113.7	2.036e5	99.44	4546	8183	1.851e4
kfSmadsComplex ($nM^{-1}min^{-1}$)	0.4670	0.01039	0.02771	2.586e-5	0.03195	3.122e-4	0.01329	1.452	1.074	2.080
krSmadsComplex ($nM^{-1}min^{-1}$)	0.2858	0.3054	4.024	2.297e-3	1.220	0.05235	0.1560	6.740	4.194	30.59
$kdeph_{pSmad2}$ (min^{-1})	999.6	1.142	0	0	0.6757	11.06	9.676	0	0	0
$kdeg_{pSmad2}$ (min^{-1})	0	0	0	0.6789	0.07010	0.9444	3.163	0.5199	0	0
v_{Smad2} ($nMmin^{-1}$)	0	0	0	0	0	0	5.714	5.714	0	0
$kdeg_{Smad2}$ (min^{-1})	0	0	0	0	0	0	0.01000	0.01000	0	0
klid ($nM^{-1}min^{-1}$)	0	6.779e-3	313.2	0	8.579	0	0	0	0	0
v_{PPM1A} ($nMmin^{-1}$)	0	0	0	0	0	0	0	7.000	0.1000	7.000
$kdeg_{PPM1A}$ (min^{-1})	0	0	0	0	0	0	0	0.07000	0.01000	0.07000
$kdeph_{PPM1A}$ (min^{-1})	0	0	0	0	0	0	0	9.356	0.3980	10.22
kPPM1A (min^{-1})	0	0	0	0	0	0	0	0	1.178e-3	0
kfPPM1A ($nM^{-1}min^{-1}$)	0	0	0	0	0	0	0	0.1819	45.63	0.3583
kbPPM1A (min^{-1})	0	0	0	0	0	0	0	41.98	7.354	282.7
krPPM1A (min^{-1})	0	0	0	0	0	0	0	1.604e-10	0	3.95e-7
kfPP ($nM^{-1}min^{-1}$)	0	0	0	0	0	0	0	82.26	0	1355
kbPP (min^{-1})	0	0	0	0	0	0	0	7.366e-11	0	5.252e-4
krPP (min^{-1})	0	0	0	0	0	0	0	0.8848	0	0.3323
kfPTEN ($nM^{-1}min^{-1}$)	0	0	0	0	0	0	0	6.312	0	4380
kbPTEN (min^{-1})	0	0	0	0	0	0	0	6.788e-10	0	3.182e-4
$kimp_{PPM1A}$ (min^{-1})	0	0	0	0	0	0	0	0.07	0	0.07
$kimp_{PP}$ (min^{-1})	0	0	0	0	0	0	0	2.378e-3	0	0.0023
$kexp_{PP}$ (min^{-1})	0	0	0	0	0	0	0	1.145e-10	0	9.471e-7
$kexp_{PTEN}$ (min^{-1})	0	0	0	0	0	0	0	3.268e-05	0	26.44
$PTEN_{Cyt}$ (nM)	0	0	0	0	0	0	0	176.3	0	176.4

Model S1 is excluded from this table because multiple fitted models (with different parameters) were selected from the structure of Model S1. The underlined values have been manually tuned. All the others were estimated numerically with MATLAB fmincon optimization. The last parameter (for PTEN) is an initial concentration. All the other parameters are rate constants.
