# Supplementary material for: The Self-Limiting Dynamics of TGF-β Signaling In Silico and In Vitro, with Negative Feedback through PPM1A Upregulation
Source: PLoS Comput Biol. 2014 Jun 5;10(6):e1003573. doi: 10.1371/journal.pcbi.1003573 (PMC4105941; doi:10.1371/journal.pcbi.1003573)
Supplement: Text S1 — The rate of receptor internalization does not affect the peak and decline of phospho-R-Smad when TGF-β is saturating. (PDF) [file pcbi.1003573.s015.pdf]

Text S1 The Rate of Receptor Internalization Does Not Affect the Peak and Decline of phospho-R-Smad When TGF-β is Saturating

Receptor internalization has multiple effects on TGF-β/Smad signaling. Internalization is a part of signal transduction because internalization promotes association of the receptor kinase with its substrate R-Smad in the cytoplasm. At the same time, receptor internalization may also act as a negative regulatory effect as it reduces the availability of receptors on the cell surface. In other systems such as EGFR pathway, receptor internalization has been shown to cause down-regulation of downstream signals [1]. Thus we studied *in silico* whether receptor internalization would have a negative regulatory effect in our system. We first tested if varying the internalization rates in Model 1 could cause a peak and decline of phospho-R-Smad. The Adaptation Index was calculated as in Eq. S1 to indicate the existence of a peak and decline. When a model has Adaptation Index=0, that means it has failed to show any decline of phospho-R-Smad.

$$\text{Adaptation Index} = \frac{[pSmad2]_{\max} - [pSmad2]_{t=24h}}{[pSmad2]_{t=24h}} \qquad \text{Eq.S1}$$

As shown in Figure S1A (blue curve), the Adaptation Index remained zero, even when the internalization rate for the ligand-receptor complex (LRC) was changed by a factor of 10^{-3} to 10^{3}. This is mainly due to early reached steady state of LRC when TGF-β dose is saturating (data not shown). Although the internalization rate could not induce Model 1 to exhibit a peak and decline of phospho-R-Smad, there may be other conditions in which internalization rates would play an important role. To find such conditions, we did perturbation analysis of all rate constants in Model 1 to see if any other parameters combined with the internalization rates could affect the Adaptation Index. We found that the Adaptation Index was most significantly increased when we decreased the production rate of T1R and increased the production rate of T2R (Figure S1C-S1D). It seems that decreasing the production rate of T1R and increasing the production rate of T2R by 10 fold in Model 1 would enhance the role of receptor internalization in the regulation of phospho-R-Smad. Indeed, inhibiting the internalization rates of LRC significantly blunted the peak of phospho-R-Smad (Figure S1B) and the internalization rates of LRC were positively correlated with the Adaptation Index (red curve, Figure S1A). However, in this scenario (low production of T1R and high production of T2R), the saturating dose of TGF-β was shifted significantly higher (Figure S1E-S1F). As shown in Figure S1F, 2ng/ml of TGF-β was no longer a saturating dose in this scenario. In contrast, we already know that 2ng/ml of TGF-β does saturate Smad signaling [2,3]. The scenario with low production of T1R and high production of T2R is not realistic for our system where 2ng/ml of TGF-β is a saturating dose, but at lower doses, receptor internalization could affect the peak and decline of phospho-R-Smad.

In summary, we have failed to identify any internalization parameters that could affect the Adaptation Index for phospho-R-Smad negative regulation, when TGF-β is saturating. In our system, we find receptor internalization does not contribute to explaining the peak and decline of phospho-R-Smad. As a result, we do not vary the parameters for receptor internalization in our analysis of negative regulatory effects.

1. Schoeberl B, Eichler-Jonsson C, Gilles ED, Muller G (2002) Computational modeling of the dynamics of the MAP kinase cascade activated by surface and internalized EGF receptors. Nat Biotechnol 20: 370-375.

2. Klipp E, Zi Z (2007) Constraint-based modeling and kinetic analysis of the smad dependent tgf-Beta signaling pathway. PLoS ONE 2: e936.

3. Zi Z, Feng Z, Chapnick DA, Dahl M, Deng D, et al. (2011) Quantitative analysis of transient and sustained transforming growth factor-beta signaling dynamics. Molecular systems biology 7: 492.
