# Supplementary material for: The Self-Limiting Dynamics of TGF-β Signaling In Silico and In Vitro, with Negative Feedback through PPM1A Upregulation
Source: PLoS Comput Biol. 2014 Jun 5;10(6):e1003573. doi: 10.1371/journal.pcbi.1003573 (PMC4105941; doi:10.1371/journal.pcbi.1003573)
Supplement: Text S2 — Extended I-Smad-mediated receptor inhibition. (PDF) [file pcbi.1003573.s016.pdf]

Text S2 Extended I-Smad-Mediated RECEPTOR INHIBITION

Our models (Model 1-7) were not capable of explaining experimental observations including the peak and decay of phospho-R-Smad, using only the three known negative regulatory effects (RECEPTOR DEGRADATION, R-SMAD DEPHOSPHORYLATION and P-SMAD DEGRADATION). The loss of type I receptor (T1R) caused by RECEPTOR DEGRADATION led us to explore degradation-independent I-Smad-mediated RECEPTOR INHIBITION effects. One possible effect we considered for negative regulation of phospho-R-Smad is dephosphorylation of the receptor-ligand complex induced by I-Smad (RECEPTOR DEPHOSPHORYLATION), which can recruit phosphatases in addition to E3-ligases [1]. Dephosphorylation would inhibit the kinase activity of the type I receptor without affecting its concentration. The other effect we considered is that I-Smad could bind to and block the active site of the type I receptor (I-SMAD ANTAGONISM). Our model of Smad activation, adopted from previously published models, includes localization effects with caveolae and endosomes, which have distinct roles in receptor endocytosis. Because I-Smad co-localizes with caveolae [2], we wanted to find a model fitting to the phospho-R-Smad data, in which I-Smad mainly associates with the ligand-receptor complex in caveolae, and in which the type I receptor is minimally lost. Figure S2 summarizes the results we got by extending the RECEPTOR INHIBITION effects to include not only RECEPTOR DEGRADATION, but also RECEPTOR DEPHOSPHORYLATION and I-SMAD ANTAGONISM. As indicated by the red box, no model was capable of maintaining the observed concentrations of total T1R, while the I-Smad was co-localized with caveolae. We were unable to construct an I-Smad-induced receptor inhibition effect that could explain the dynamics of phospho-R-Smad.

1. Shi W, Sun C, He B, Xiong W, Shi X, et al. (2004) GADD34-PP1c recruited by Smad7 dephosphorylates TGFbeta type I receptor. J Cell Biol 164: 291-300.

2. Di Guglielmo GM, Le Roy C, Goodfellow AF, Wrana JL (2003) Distinct endocytic pathways regulate TGF-beta receptor signalling and turnover. Nat Cell Biol 5: 410-421.
