# Supplementary material for: The Self-Limiting Dynamics of TGF-β Signaling In Silico and In Vitro, with Negative Feedback through PPM1A Upregulation
Source: PLoS Comput Biol. 2014 Jun 5;10(6):e1003573. doi: 10.1371/journal.pcbi.1003573 (PMC4105941; doi:10.1371/journal.pcbi.1003573)
Supplement: Text S3 — Sensitivity analysis. (PDF) [file pcbi.1003573.s017.pdf]

Text S3 Sensitivity Analysis

We performed sensitivity analysis on the parameters of Model 5 to determine which proteins levels would most sensitively reflect the behavior of degradation effects in the model. We computed the local sensitivities of all species to the RECEPTOR DEGRADATION and P-SMAD DEGRADATION effects. This was done by perturbing the parameters related to these effects individually, and computing the effect of the perturbations on the protein levels. The parameters were perturbed by a spectrum of relative changes (from 10^{-4} to 10^{2}) with the expectation that changes within 10-fold would be most relevant. The sensitivities (Figure S3) indicate that the total type I receptor and total R-Smad levels have higher sensitivity than other proteins did, to the RECEPTOR DEGRADATION and P-SMAD DEGRADATION effects, respectively. This confirms our choice of total T1R levels and total R-Smad levels for testing our hypotheses about RECEPTOR DEGRADATION and P-SMAD DEGRADATION.
