# Supplementary material for: The Self-Limiting Dynamics of TGF-β Signaling In Silico and In Vitro, with Negative Feedback through PPM1A Upregulation
Source: PLoS Comput Biol. 2014 Jun 5;10(6):e1003573. doi: 10.1371/journal.pcbi.1003573 (PMC4105941; doi:10.1371/journal.pcbi.1003573)
Supplement: Text S4 — PPM1A upregulation could possibly be a slow-mode effects. (PDF) [file pcbi.1003573.s018.pdf]

Text S4 PPM1A UPREGULATION Could Possibly Be a Slow-Mode Effect

A model with R-SMAD DEPHOSPHORYLATION and the phosphatase (PPM1A) upregulated by nuclear phospho-R-Smad (PPM1A UPREGULATION BY EXPRESSION, Model S2) is able to fit to both phospho-R-Smad data after short and long exposure of TGF-β (Figure S4A-B). Model S2 also predicted unchanged levels of T1R and total R-Smad, which is consistent with our experiments (Figure S4C-D).
