# Supplementary material for: The Self-Limiting Dynamics of TGF-β Signaling In Silico and In Vitro, with Negative Feedback through PPM1A Upregulation
Source: PLoS Comput Biol. 2014 Jun 5;10(6):e1003573. doi: 10.1371/journal.pcbi.1003573 (PMC4105941; doi:10.1371/journal.pcbi.1003573)
Supplement: Text S5 — Model-based prediction of PPM1A Stabilization dynamics. (PDF) [file pcbi.1003573.s019.pdf]

Text S5 Model-based Prediction of PPM1A Stabilization Dynamics

Previous work does not indicate how PPM1A might be upregulated by TGF-β, but the speed of the effect suggests post-translational regulation (such as enhanced stability) or post-transcriptional regulation. PTEN has been shown in fibroblasts to associate with PPM1A and to protect PPM1A from proteasomal degradation [1]. In the fibroblast context, TGF-β caused decreased levels of PPM1A, and caused dissociation of PTEN from phospho-R-Smad. It is possible that in our HaCaT system (keratinocytes), TGF-β could cause the opposite effect, upregulation of PPM1A, but also by regulating the association with PTEN. In HaCaT cells, TGF-β was reported to cause *increased* association between PTEN and phospho-R-Smad [2], not the dissociation observed in fibroblasts. We therefore propose Model 9 (specific to HaCaT cells), in which TGF-β would induce stabilization of PPM1A by PTEN. In this model, we halted degradation of PPM1A whenever it was associated with PTEN, but PTEN was not allowed to form a complex directly with PPM1A, (i.e., in the absence of TGF-β stimulation). Rather, a ternary complex of pSmad2:PTEN:PPM1A could form, and a PTEN:PPM1A complex could be released from the ternary complex. This recapitulates the observation that PTEN-Smad2 association is dependent on TGF-β stimulation [2]. In this model, we required the upregulation of PPM1A protein to match the fast kinetics observed, but we also wanted the increased phosphatase activity against phospho-R-Smad to match the observed phospho-R-Smad dynamics. We do not know whether PPM1A activity, conformation, or localization would be changed by binding to PTEN. It is possible that PTEN-PPM1A association could cause some transient sequestration of PPM1A activity, which might delay the functional impact of PPM1A upregulation. In Model S3, we assumed that unbound PPM1A is rapidly imported into the nucleus, but PPM1A bound to PTEN has slow import into the nucleus. Because phospho-R-Smad accumulates in the nucleus, delayed nuclear import of PPM1A during PTEN-mediated stabilization would delay the functional impact of PPM1A activity, relative to the total PPM1A levels. Complete reaction equations and parameters appear in the Supporting Information. Model S3 successfully recapitulated the experimentally observed dynamics of phospho-R-Smad (Figure S5A); the fast kinetics of PPM1A upregulation (Figure S5B); and the unchanged levels of T1R and total R-Smad (Figure S5C-D).

1. Bu S, Kapanadze B, Hsu T, Trojanowska M (2008) Opposite effects of dihydrosphingosine 1-phosphate and sphingosine 1-phosphate on transforming growth factor-beta/Smad signaling are mediated through the PTEN/PPM1A-dependent pathway. J Biol Chem 283: 19593-19602.

2. Hjelmeland AB, Hjelmeland MD, Shi Q, Hart JL, Bigner DD, et al. (2005) Loss of phosphatase and tensin homologue increases transforming growth factor beta-mediated invasion with enhanced SMAD3 transcriptional activity. Cancer research 65: 11276-11281.
