# Supplementary material for: The Self-Limiting Dynamics of TGF-β Signaling In Silico and In Vitro, with Negative Feedback through PPM1A Upregulation
Source: PLoS Comput Biol. 2014 Jun 5;10(6):e1003573. doi: 10.1371/journal.pcbi.1003573 (PMC4105941; doi:10.1371/journal.pcbi.1003573)
Supplement: Text S6 — Transient and sustained signaling in the TGF-β signaling pathway. (PDF) [file pcbi.1003573.s020.pdf]

Text S6 Transient and Sustained Signaling in the TGF-β Signaling Pathway

Our final model with PPM1A STABILIZATION (Model 8) is also capable of producing the same dose-response behavior in short-term and long-term signals as shown by Klipp et al. [1]. The phospho-R-Smad concentration at 45 min increased when the dose of TGF-β did (Figure S6A). But if the dose of TGF-β were high enough, the phospho-R-Smad concentration would be saturated at 45 min. The concentration of phospho-R-Smad did not remain elevated at 24 hr after TGF-β treatment unless the TGF-β dose exceeds a certain threshold (Figure S6B). This shows an ultrasensitive signaling response to TGF-β dose. We speculate that the saturation of the signal is mainly due to saturated TGF-β receptor, since R-Smad was not saturated (only 30% of the R-Smad was phosphorylated). If the receptors were saturated, then the dose of TGF-β only affects the duration, but not the intensity of the signal in the long-term (Figure S6C). This suggests that the reason for the decay of phosphorylated R-Smad with saturating TGF-β dose is down-stream regulatory effects, but not the consumption of TGF-β.

1. Zi Z, Feng Z, Chapnick DA, Dahl M, Deng D, et al. (2011) Quantitative analysis of transient and sustained transforming growth factor-beta signaling dynamics. Molecular systems biology 7: 492.
