# Supplementary material for: The Self-Limiting Dynamics of TGF-β Signaling In Silico and In Vitro, with Negative Feedback through PPM1A Upregulation
Source: PLoS Comput Biol. 2014 Jun 5;10(6):e1003573. doi: 10.1371/journal.pcbi.1003573 (PMC4105941; doi:10.1371/journal.pcbi.1003573)
Supplement: Text S7 — Evaluation of best-fits of all models. (PDF) [file pcbi.1003573.s021.pdf]

Text S7 Evaluation of Best-fits of All Models

The goodness-of-fit of models was evaluated by the Sum of Squared Error (SSE) of simulated species levels to experimental data. We fitted our models to the dynamics of phospho-R-Smad primarily. Model 8, S2 and S3 were fitted too PPM1A levels as well. Figure S7 shows the SSE of the best-fits of all models except Model S1). For Model S1, not only the best-fit but a set of fitted Model S1 (SSE < 5000) were analyzed. Please note that we were seeking qualitatively fitted models (e.g. peak and decline of phospho-R-Smad). Small differences between SSE values may not determine the which model fits the data better. Thus, when we examined the parameter estimation results, we examined a set of models with low SSE values. Because their simulated dynamics were similar, we chose the best-fit as the one with the lowest SSE value.
