# Supplementary material for: The Self-Limiting Dynamics of TGF-β Signaling In Silico and In Vitro, with Negative Feedback through PPM1A Upregulation
Source: PLoS Comput Biol. 2014 Jun 5;10(6):e1003573. doi: 10.1371/journal.pcbi.1003573 (PMC4105941; doi:10.1371/journal.pcbi.1003573)
Supplement: Text S8 — Modeling cycloheximide treatment. (PDF) [file pcbi.1003573.s022.pdf]

Text S8 Modeling Cycloheximide Treatment

There is one more test data set we can use to validate our final model (Model 8). The dataset is originally from Inman *et al.* [49] and it shows the nuclear phospho-R-Smad levels in HaCaT cells with and without cycloheximide treatment. This dataset has also been used in [50] for validation of their model. As shown in Figure S8A, phospho-R-Smad level decreases faster in the group pretreated with cycloheximide than that in the control group. Since our model was not trained toward any nuclear fraction of phospho-R-Smad, we compared our simulated overall phospho-R-Smad levels to this dataset as Vilar *et al.* did [50]. Because cycloheximide has a global effect of inhibiting protein production, the effect of cycloheximide was simulated as all production rates (including the production rates for T1R, T2R, Smad2 and PPM1A) decreased to half of their value 30 min before TGF-β treatment (cycloheximide was pretreated for 30min in [50]). The prediction of our Model 8 was able to qualitatively reproduce the difference between the group with cycloheximide treatment (red curve) and the group without cycloheximide treatment (blue curve). We note that our simulation did not match the dataset at early time points (<1hr). This is probably because we did not fit our model to data points earlier than 1hr. Vilar *et al.* also suggested that more accurate modeling framework for the receptor trafficking could help the model to fit the early dynamics better [50].
